# Unity in diversity: a survey of muscular systems of ctenostome Gymnolaemata (Lophotrochozoa, Bryozoa)

**DOI:** 10.1186/s12983-018-0269-6

**Published:** 2018-06-07

**Authors:** Thomas F. Schwaha, Andreas Wanninger

**Affiliations:** University of Vienna, Department of Integrative Zoology, Althanstraße 14, 1090 Vienna, Austria

**Keywords:** Muscle evolution, Hydrostatic system, Ctenostomata, Phalloidin staining, Confocal laser scanning microscopy, Lophophore

## Abstract

**Background:**

Myoanatomical studies of adult bryozoans employing fluorescent staining and confocal laser scanning microscopy (CLSM) have been chiefly conducted on freshwater bryozoans. The diversity of muscular systems in the marine bryozoans is currently not well known with only two species being studied in more detail. The aim of this study is to unravel the diversity of muscle systems of 15 ctenostome bryozoans by phalloidin-coupled fluorescence stainings combined with CLSM.

**Results:**

In general, the myoanatomy of the selected ctenostomes shows significant similarities and consists of 1) muscles associated with the body wall, 2) apertural muscles, 3) lophophoral muscles, 4) tentacle sheath muscles, 5) digestive tract muscles and 6) the prominent retractor muscles. Differences are present in the arrangement of the apertural muscles from generally three muscles sets of four bundles, which in some species can be partially reduced or modified into a bilateral arrangement. The cardiac region of the digestive tract shows a distinct sphincter in four of the six studied clades. In some cases the cardiac region forms a prominent proventriculus or gizzard. Tentacle sheath muscles in victorelloideans and walkerioideans are arranged diagonally and differ from the simple longitudinal muscle arrangements common to all other taxa. Lophophoral base muscles consist of four sets that vary in the size of the sets and in the shape of the inner lophophoral ring, which either forms a complete ring or separate, intertentacular muscle bundles. The stolon-forming walkeridiodean ctenostomes show prominent transverse muscles in their stolons. These are always present in the shorter side stolons, but their occurrence in the main stolon seems to depend on the colony form, being present in creeping but absent in erect colony forms.

**Conclusions:**

This study represents the first broad survey of muscular systems in adult ctenostome bryozoans and shows a certain degree of conservation in a series of diverse colony forms belonging to five major clades. However, several myoanatomical features such as the cardiac sphincter, basal (possibly transitory) cystid muscles, tentacle sheath muscles or apertural muscle arrangement vary across taxa and thus show a high potential for the assessment of character evolution within ctenostomes. As such, this study represents an essential contribution towards determining and reconstructing the character states of the bryozoan ground pattern once a reliable phylogenetic tree of the whole phylum becomes available.

**Electronic supplementary material:**

The online version of this article (10.1186/s12983-018-0269-6) contains supplementary material, which is available to authorized users.

## Background

Bryozoa is a phylum of colonial invertebrate animals with 6000 recent and 15.000 fossil species described [1]. Most bryozoans are colonial and consist of repetitive modules of individuals, so-called zooids, which constitute the colony. Each zooid consists of a protective body wall called the cystid and a polypide, consisting of the soft body parts. Within the polypide are the ciliated tentacle crown (the lophophore), u-shaped digestive tract as well as associated muscles, associated neuronal elements and gonads where present. Typical for all bryozoans is the retraction process; a defensive response where the polypide is retracted into the cystid for protection [2, 3]. Eversion of the tentacle crown is achieved by an increase in coelomic pressure [3, 4].

Three different taxa of bryozoans are currently recognized: Phylactolaemata, Stenolaemata and Gymnolaemata [3]. Among other features, each of these groups is characterized by its cystid structure and thus a respective protrusion mechanism [4]. The phylactolaemate cystid is unmineralized and flexible which allows contraction of the regular body wall musculature to increase coelomic pressure upon contraction thereby everting the polypide [5, 6]. Stenolaemates have calcified and rigid cystids which are incompressible. Consequently, the coelomic epithelium is detached from the body to form a so-called membranous sac around the polypide which allows contraction by annular ring muscles in its epithelial lining for polypide eversion (see [3, 4, 7]).Gymnolaemata is the most species-rich and diverse taxon of bryozoans, comprising over 5000 species. The gymnolaemate cystid can be either uncalcified and flexible such as those of the paraphyletic “Ctenostomata” [8] or partially or fully calcified such as those of the Cheilostomata [9]. Both these groups possess parietal muscles derived from putative ancestral body-wall musculature, which they use for polypide eversion [3, 9]. Most of these protrusion mechanisms were already described in the 19th century by light-microscopy combined with histological sectioning (e. g. [10–12]). Information on the other muscular systems (e.g., the digestive tract or lophophore) are scarce and mainly consists of a few whole-mount staining analyses [13–15] or ultrastructural studies that focused on specific organ systems (e.g. [16–20]).

The application of confocal laser scanning microscopy in the past decade has yielded new insights into the neuro-muscular system of larval and adult bryozoans (e.g. [6, 21–36]). Adult muscular systems have only been studied in a few phylactolaemate and ctenostome species [6, 28, 30, 35]. These studies demonstrated distinct differences in the digestive tract and lophophore muscle systems between phylactolaemates and ctenostomes. However, knowledge of the broader diversity of bryozoan muscular systems is lacking as only two of the six major clades have been studied [8, 37]. The aim of the current work was to investigate the diversity of ctenostome muscular systems by analyzing five of the six major ctenostome clades. The results presented here will be of substantial importance for the reconstruction of the muscular ground pattern of ctenostome bryozoans.

## Methods

Fifteen species representing five ctenostome major clades were collected over a period of a decade from various localities (Sweden, Croatia, Thailand, Orkney Islands, Japan, Maledives, Table 1). All samples were fixed in 4% paraformaldehyde in 0.1 M PB (pH = 7.4) for 1 h at room temperature followed by three washes in PB. With the exception of *Triticella flava*, all specimens were generally fixed in a retracted condition. The samples were then stored in 0.1 M PB containing 0.1% NaN_3_. Staining of the muscular system was conducted with phalloidin or phallacidin coupled to AlexaFluor 488 or Bodipy FL phallacidin (Invitrogen, Molecular Probes, Eugene, OR, USA) at a concentration of 1:40–1:60 in PB containing 1–4% Triton-X-100, for some samples 2% DMSO was added for enhanced permeabilization. Staining was performed overnight followed by counterstaining of nuclei with DAPI (1:200). Excessive staining solution was eluted with several rinses in PB. The samples were mounted in Fluoromount G (Southern Biotech, Birmingham, AL, USA). Analysis of the samples was performed on a Leica SP2 or a SP5 II confocal laser scanning microscope (Leica Microsystems, Wetzlar, Germany). Depending on the objective, optical z-stacks of the samples were acquired with a thickness of 0.4-1 μm step size. Data analysis was conducted with FIJI (www.fiji.sc, [38]) or Amira 6.3 (FEI, Hillsboro, Oregon, USA). Several zooids of each species were analysed. However, for *Ascorhiza* cf. *mawatarii*, only a single colony was available for the analysis. Intraspecfic variation of various bryozoans species is very limited or missing (Schwaha, pers. observation of 50 bryozoan species).

**Table 1:** List of ctenostome bryozoans here studied

Superfamily	Family	Species
Alcyonidioidea	Alcyonidiidae	*Alyconidium gelatinosum*
Alcyonidioidea	Alcyonidiidae	*Alcyonidium diaphanum*
Alcyonidioidea	Clavoporidae	*Ascorhiza* cf. *mawatarii*
Arachnidioidea	Nolellidae	*Nolella dilatata*
Arachnidioidea	Nolellidae	*Nolella* sp.
Arachnidioidea	Nolellidae	*Nolella*(?) sp.
Paludicelloidea	Palludicellidae	*Paludicella articulata*
Vesicularioidea	Vesiculariidae	*Amathia semiconvoluta*
Vesicularioidea	Vesiculariidae	*Amathia (Zoobotryon) verticillata*
Vesicularioidea	Buskiidae	*Cryptopolyzoon wilsoni*
Victorelloidea	Victorellidae	*Victorella pavida*
Walkerioidea	Mimosellidae	*Mimosella gracilis*
Walkerioidea	Mimosellidae	*Mimosella* sp. erect
Walkerioidea	Aeverilliidae	*Aeverillia setigera*
Walkerioidea	Triticellidae	*Triticella flava*

## Results and discussion

### Description and comparison of different ctenostome muscle systems

**Fig. 1:**
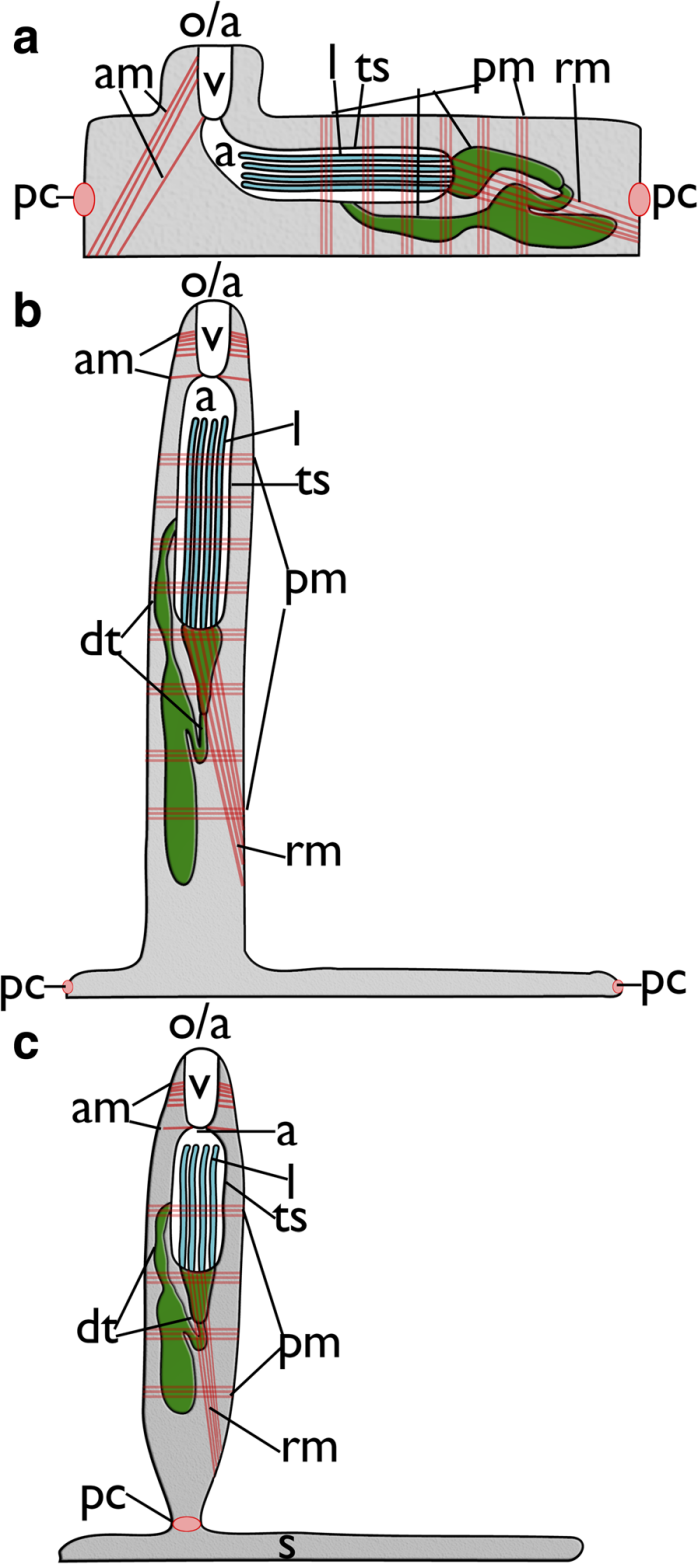
Schematic representation of ctenostome bryozoans including main muscle system. **a** Simple colony morphology as e.g. in *Paludicella* or *Alcyonidium* **b** Zooids with elongated peristomes such as found in the genus *Nolella*. **c** stolon-bearing colonies with stolonal kenozooids that interconnect feeding zooids (autozooids) with each other. Abbreviations: a – atrium, am – apertural muscles, dt – digestive tract, l – lophophore, o/a – orifice/aperture of the zooid, pc – pore complex, pm – parietal muscles, rm – retractor muscle, s - stolon, ts – tentacle sheath, v – vestibulum

Ctenostome bryozoans show three main distinct zooidal morphologies: simple (often uniserial) zooids with short or little pronounced peristomial area (e.g. where the lophophore is protruded) on the frontal side (Fig. 1a), zooids with distinct elongated peristomes (Fig. 1b) and stolonal forms (Fig. 1c).

**Fig. 2:**
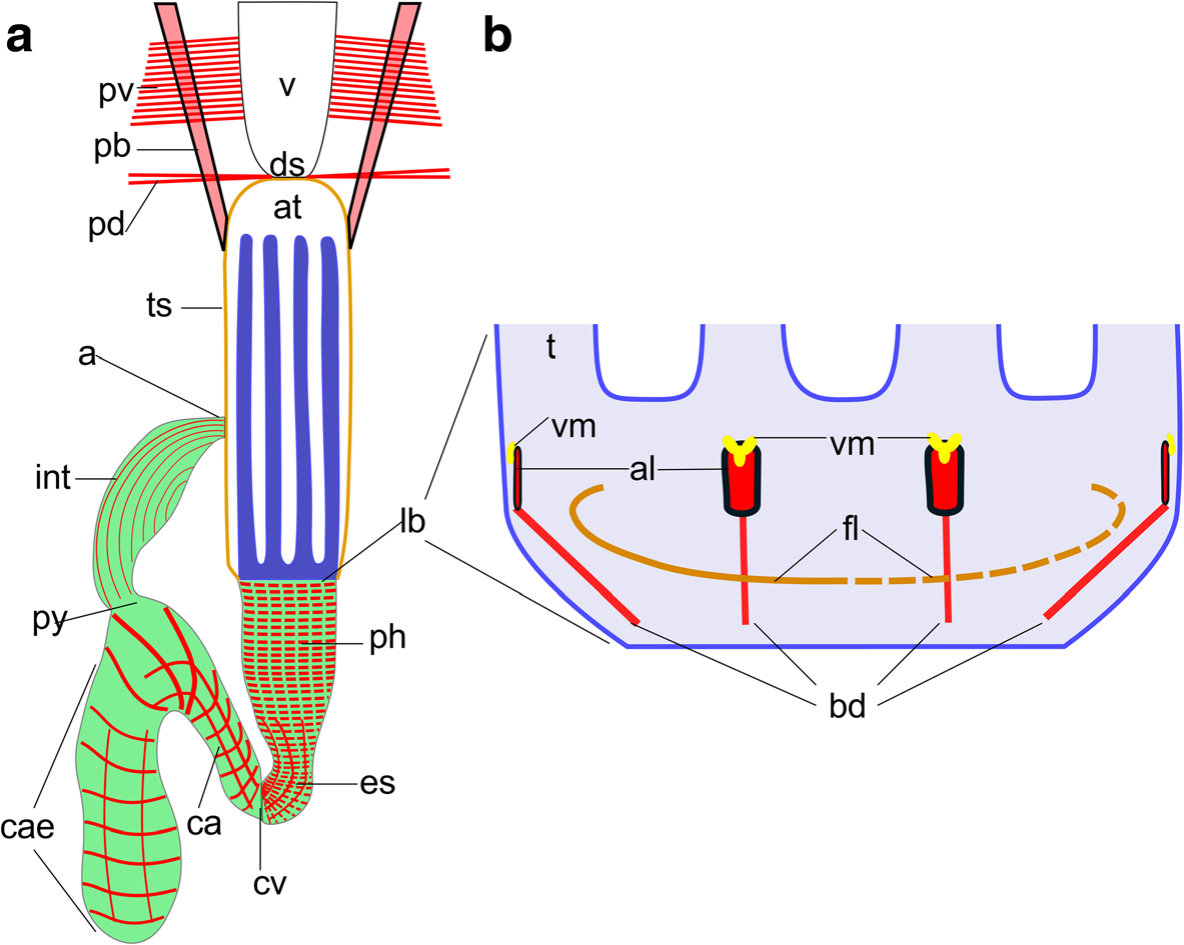
Schematic overview of muscular systems in ctenostome bryozoans. **a** General outline of a retracted polypide showing the apertural area with the vestibulum followed by the atrium, which is bordered by the tentacle sheath (orange) and harbors the retracted lophophore (blue). The digestive tract (green) has prominent striated muscles in the foregut (pharynx, esophagus), whereas the remaining mid- and hindgut have sparse, smooth muscles. **b** Detail of the lophophoral base muscles showing the four different sets of muscles present. The inner/frontal muscle ring is shown continuous on the left side and discontinuous on the right (see text for details). Abbreviations: a – anus, al - abfrontal lophophoral base muscle, at – atrium, bd - buccal dilatator, ca – cardia, cae – caecum, cv – cardiac valve, ds – diaphragmatic sphincter, es – esophagus, fl - frontal lophophoral base muscle, int – intestine, lb – lophophoral base, pb – parietovaginal band, pd – parietodiaphragmatic muscle, ph – pharynx, py – pylorus, pv – parietovestibular muscles, t – tentacle, ts – tentacle sheath, v – vestibulum

The main muscular systems of bryozoans can be divided into six different categories (see Fig. 2): 1) the musculature of the body wall and associated musculature, 2) the apertural musculature. The latter are essential for the closing and opening the aperture/orifice, i.e. the region where the polypide retracts into the cystid; 3) the tentacle sheath muscles, 4) the muscles associated with the digestive tract, 5) the musculature of the lophophore, which includes muscles of the tentacles as well as the lophophoral base, 6) the prominent retractors that insert at the lophophoral base. Details on each species are summarized as supplementary table (Additional file 1).

#### Body-wall muscles and their derivatives

**Fig. 3:**
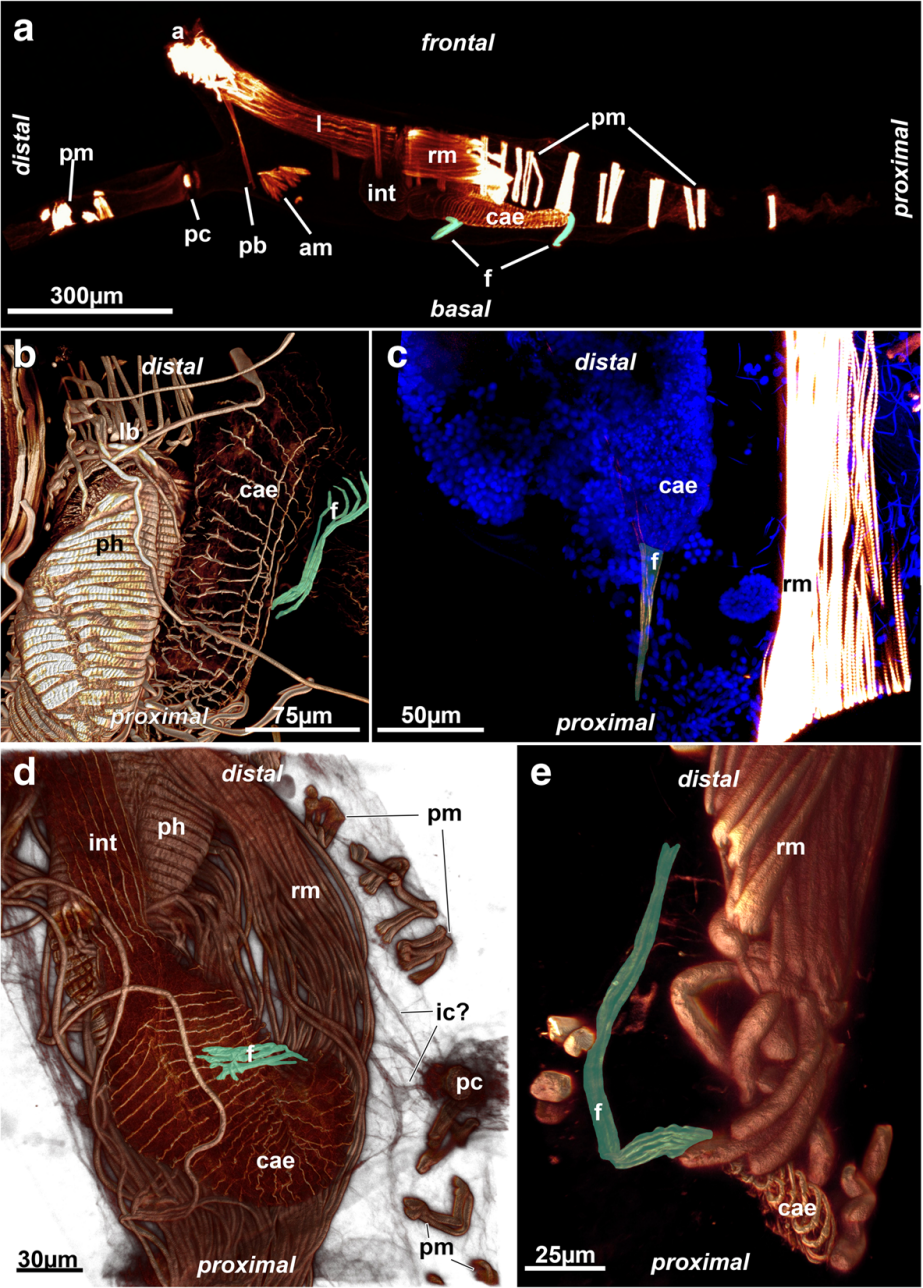
Muscular funiculus in ctenostomes. The funiculus musculature is highlighted in green. **a** Paludicelloidea, *Paludicella articulata*. Lateral view of an entire, partially retracted zooid with its lophophore and parts of the caecum on the basal side. Note the two funiculi, one distally and another one proximally positioned, that attach parallel to the caecum. **b** Alcyonidioidea. *Alcyonidium diaphanum,* close-up of the digestive tract and a muscular funiculus that attaches to the caecum. **c** Walkerioidea, *Triticella flava*. Muscular funiculus extending from the proximal tip of the caecum to the body wall. **d** Victorelloidea, *Victorella pavida*. Close-up of the digestive tract showing a muscular funiculus attaching to the proximal caecum. Note that the caecum is slightly bent. Also note thin strands projecting from the pore complex towards the polypide. **e** Arachnidioidea, *Nolella* sp., showing an elongated thin funiculus extending from the caecum. Abbreviations: a – aperture, am – apertural muscle, cae – caecum, f – funiculus, ic – possible interconnecting strands, int – intestine, l – lophophore, lb – lophophoral base, pb – parieto-vaginal band, pc – pore complex, ph – pharynx, pm – parietal muscles, rm – retractor muscle

**Fig. 4:**
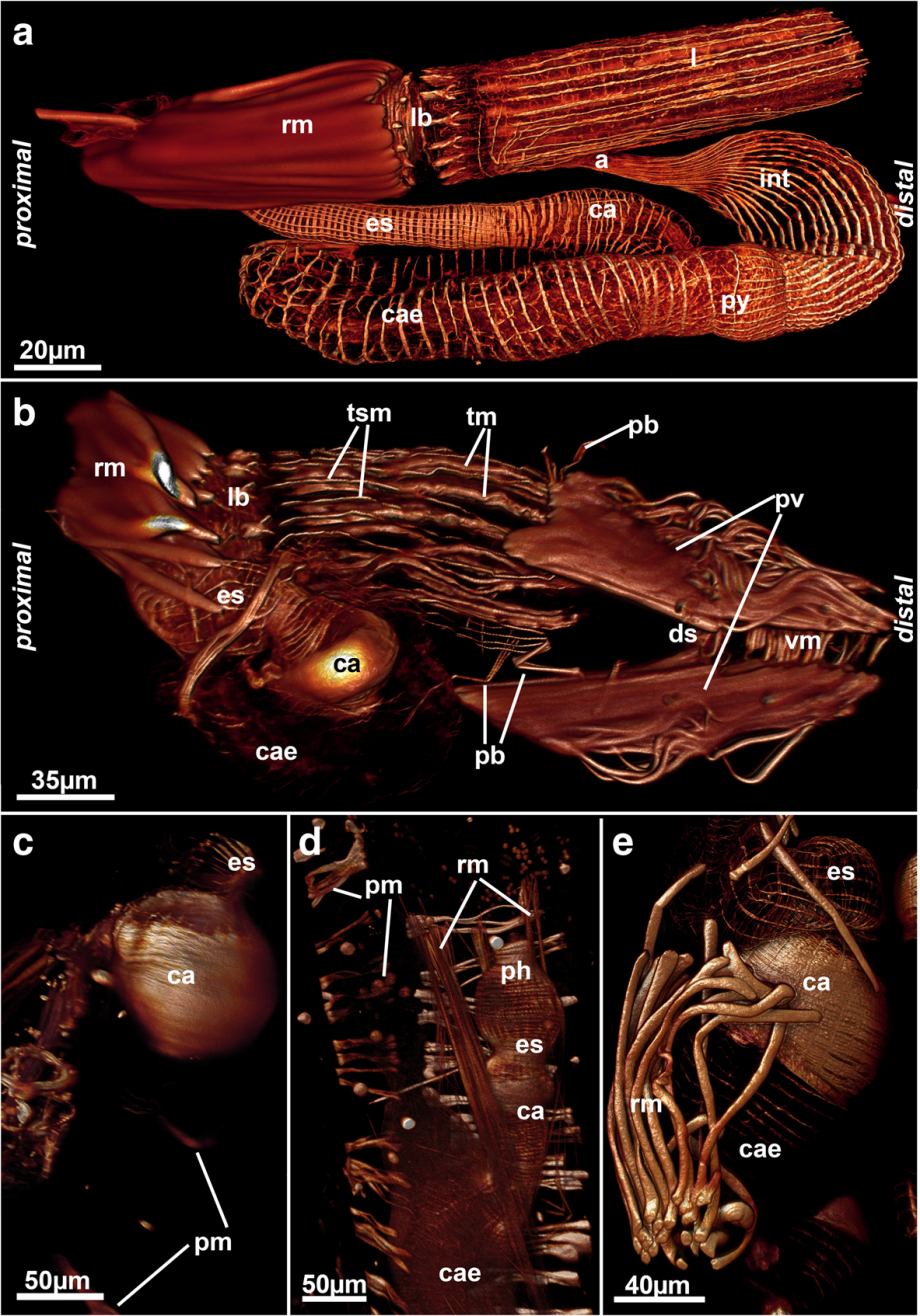
Myoanatomy of the digestive tract of selected ctenostomes. **a** Paludicelloidea, *Paludicella articulata.* Dissected zooid and overview of the main regions of the gut and its musculature. Volume rendering. **b** Vesicularioidea, *Cryptopolyzoon wilsoni*. Lateral view of a zooid showing a distinct prominent cardiac proventriculus. Volume rendering. **c** Arachnidioidea, *Nolella dilata*. Detail of the cardia showing more prominent circular musculature. Volume rendering. **d** Arachnidioidea, *Nolella*(?) sp. Detail of the gut with distinct concentration of cardiac circular musculature. Volume rendering. **e** Vesicularioidea, *Amathia verticillata,* showing the prominent cardiac sphincter. Volume rendering. Abbreviations: a – anus, ca – cardia, cae – caecum, ds – diaphragmatic sphincter, es – esophagus, int – intestine, l – lophophore, lb – lophophoral base, pb – parieto-vaginal band, ph - pharynx, pm – parietal muscles, py – pylorus, pv – parieto-vestibular muscles, rm – retractor muscle, tm – tentacle muscles, tsm – tentacle sheath muscles, vm – vestibular wall muscles

**Fig. 5:**
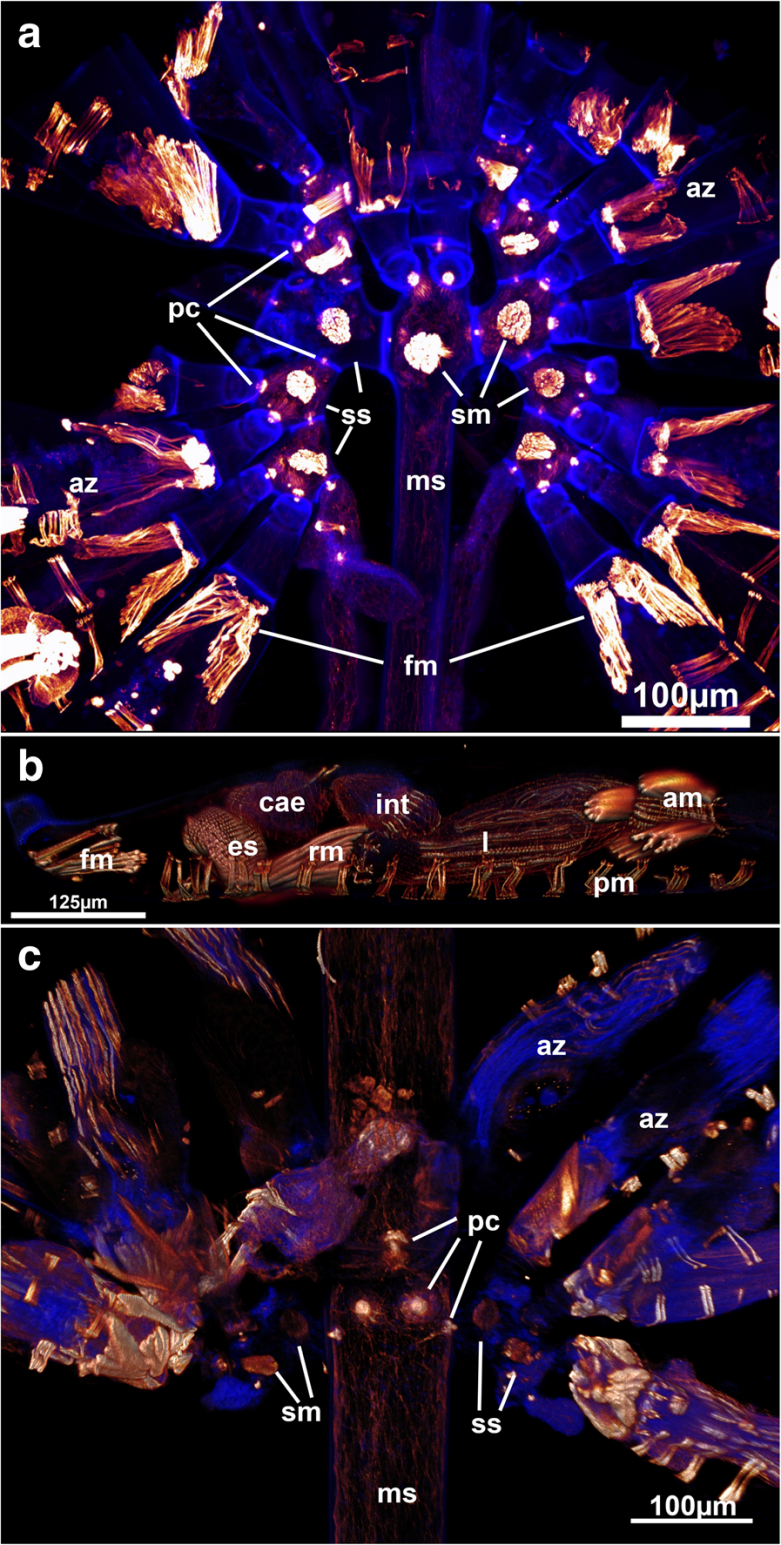
Muscular system in the walkerioidean genus *Mimosella*. **a** *Mimosella verticillata* showing the main and side stolons and their associated stolonal transverse muscle. Note the paired flexor muscles at the base of autozooids attached to the side stolons. **b** Lateral view of a single zooid of *M. verticillata* showing the position of the flexor muscle close to the stalk-like attachment area of the zooid to the stolon (left side). **c** *Mimosella* sp. erect form. Similar colonial arrangement such as found in (A), but erect colonial form compared to the creeping *M. verticillata* in (A). Note the thicker main stolon and absence of the stolonal transversal muscle in the main stolon. Abbreviations: am – apertural muscles, az – autozooid, cae –caecum, es – esophagus, fm – flexor muscles, int – intestine, ms – main stolon, pc – pore complex, pm – parietal muscles, rm – retractor muscle, sm – stolonal transverse muscle, ss – side stolons

**Fig. 6:**
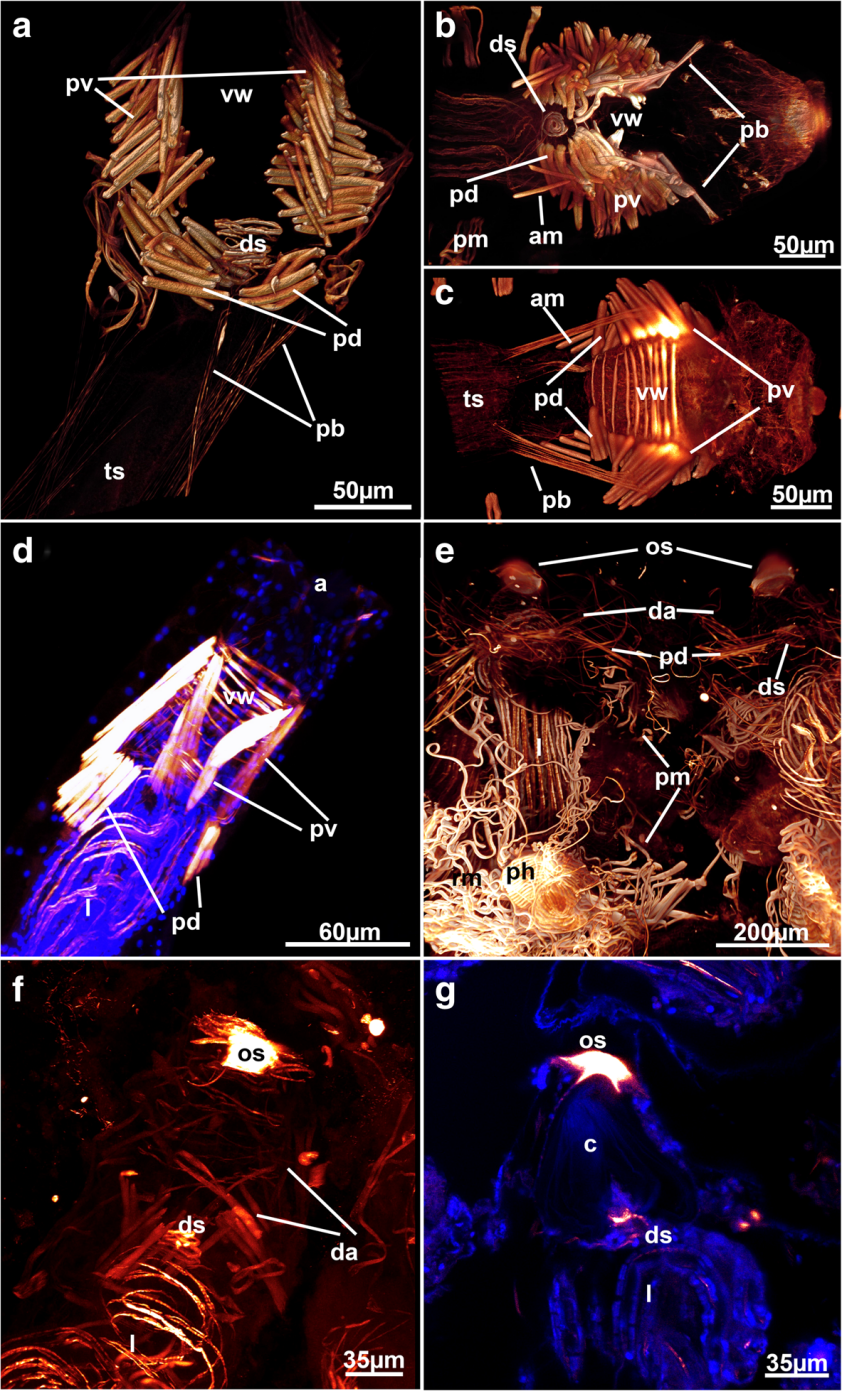
Apertural muscles of selected ctenostome bryozoans. **a** Arachnidioidea, *Nolella dilatata*. Lateral view of the apertural muscles. Volume rendering. (B&C) Paludicelloidea, *Paludicella articulata*. Volume rendering. View of the apertural area, **b** Frontal side. **c** Basal side. **d** Walkerioidea, *Mimosella* sp., erect form. Lateral view, Maximum intensity projection. **e** Alcyonidioidea, *Alcyonidium diaphanum*. Overview of two adjacent zooids. Note the prominent orificial sphincter and the remaining diffuse apertural musculature. (F&G) Alcyonidioidea, *Ascorhiza mawatarii*. **f** Lateral view of the musculature of the aperture showing a prominent orifical sphincter and less conspicuous diffuse apertural muscles. Maximum intensity projection. **g** Optical section of the apertural area showing the diaphragmatic sphincter with the collar just underneath the orificial sphincter. Abbreviations: a – aperture, am – additional apertural muscle, c – collar, da – diffuse apertural muscle, ds – diaphragmatic sphincter, l - lophophore, os – orificial sphincter, pb – parieto-vaginal bands, pd – parieto-diaphragmatic muscle, ph – pharynx, pm – parietal muscle, pv – parieto-vestibular muscle, rm –retractor muscle, ts – tentacle sheath, vw – vestibular wall muscle

**Fig. 7:**
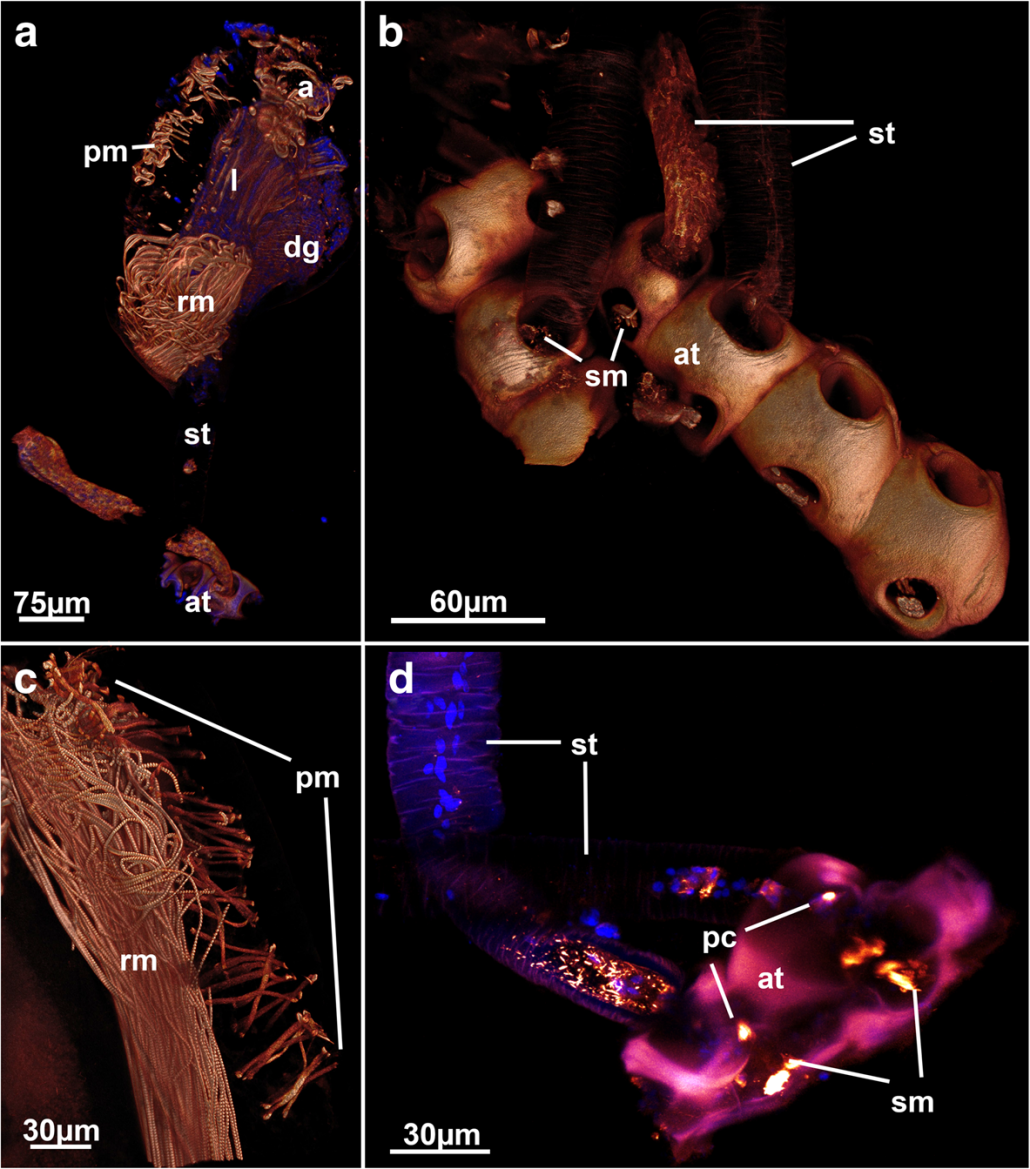
Muscular details of the walkerioidean *Triticella flava*. **a** General overview of the myoanatomy of a single zooid. Note the thin stalk that attaches the zooid to the small attachment disc. Volume rendering. **b** Detail of attachment sites of a colony. Note the strong autofluorescence of the dome-shaped cuticle and small portions of basal muscles inside. Volume rendering. **c** Detail of the parietal muscle, which is restricted to one side of the zooid. Note also the striated retractor muscles. Volume rendering. **d** Detail of the attachment area of a colony showing two stalks attached to the highly autofluorescent cuticle of the attachment area. Note the short basal stolonal muscles in the attachment area. Maximum intensity projection. Abbreviations: a – aperture, at – attachment area, dg – digestive tract, l – lophophore, pc – pore complex, pm – parietal muscle, rm – retractor muscle, sm – stolonal transverse muscle, st – stalk

A series of parietal muscles that traverse the body cavity laterally are generally present in ctenostome bryozoans (Figs. 1, 3a,d, 4d, 5b). They extend from the basal or lateral side of the zooids towards the frontal side of each zooid. The parietal muscles are a series of transverse muscle bundles (Figs. 1, 3a,d, 4d, 5b and 6e), which was also reported in most previous descriptions of ctenostomes (e.g. [3, 9, 11, 15]). In the walkerioidean *Triticella flava*, parietal musculature is not paired and restricted to one lateral side of the zooid (Fig. 7a and c). It seems that the insertion of these muscles corresponds to the area of the frenaculum, a distinct thick, cuticular rim diagnostic of the genus [39]. In the alcyonidioid *Ascorhiza* cf. *mawatarii*, no parietal muscles could be observed, but material of this genus limited to a single colony. Parietal muscles consist of a smooth, non-striated fiber type [3, 9].

**Fig. 8:**
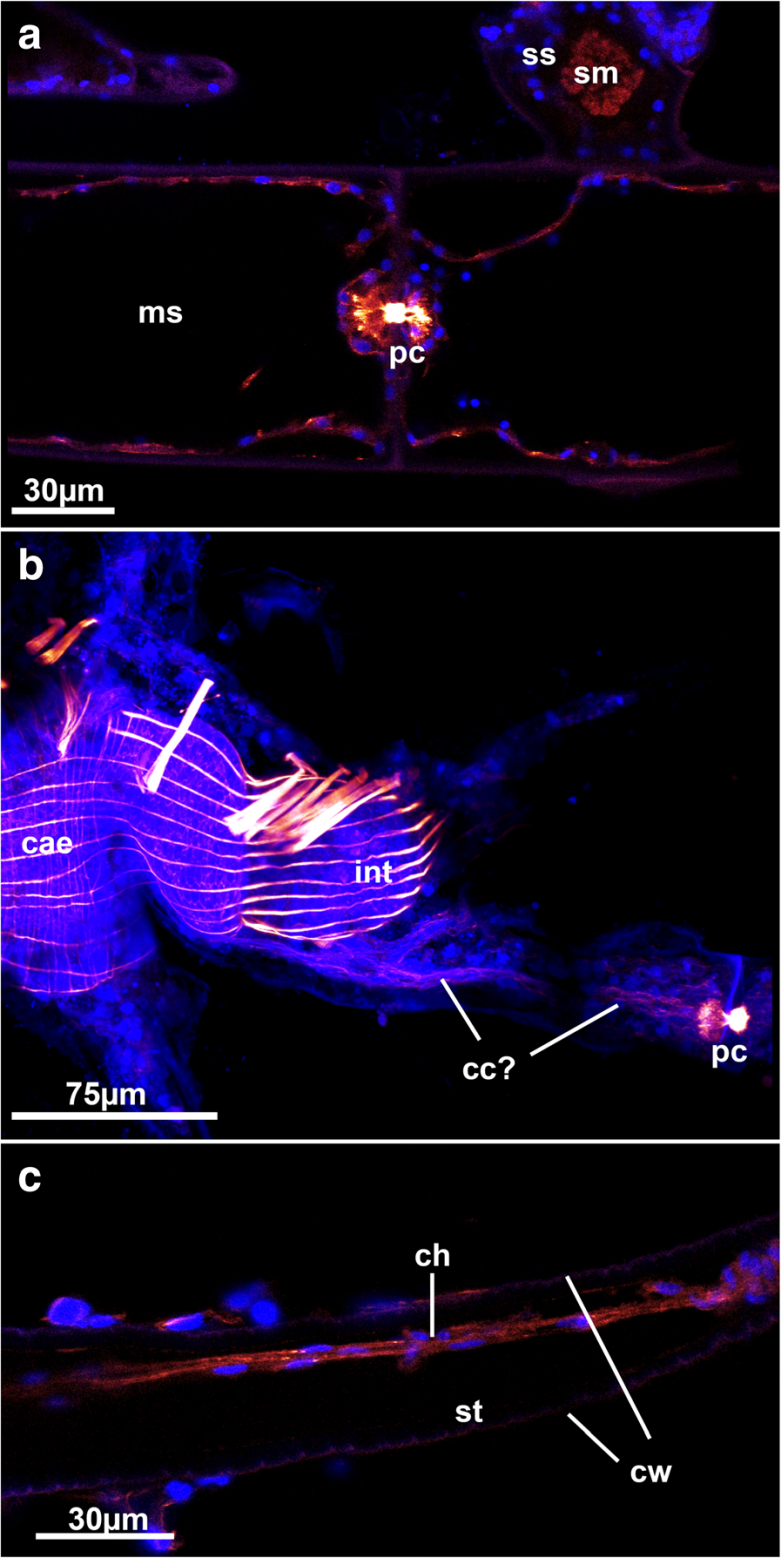
Pore complex and putative interconnecting strands in ctenostomes. **a** Pore complex of the walkerioidean *Mimosella* sp., erect form. The inner part of the pore complex shows distinct f-actin concentration. Note the lack of strands or tissues in association with the pore complex. Optical section. **b** Pore complex in the arachnidioidean *Nolella dilatata* showing cord-like structures from the pore towards the polypide. Maximum intensity projection (**c**) Tissue cord in the basal stalk of the walkerioidean *Triticella flava*. Note the lack of nuclei adjacent to the cystid wall. Maximum intensity projection. Abbreviations: cae – caecum, cc – connective cord, ch – tissue cord, int – intestine, ms – main stolon, pc – pore complex, sm – stolonal transverse  muscle, ss – side stolon, st – stalk

**Fig. 9:**
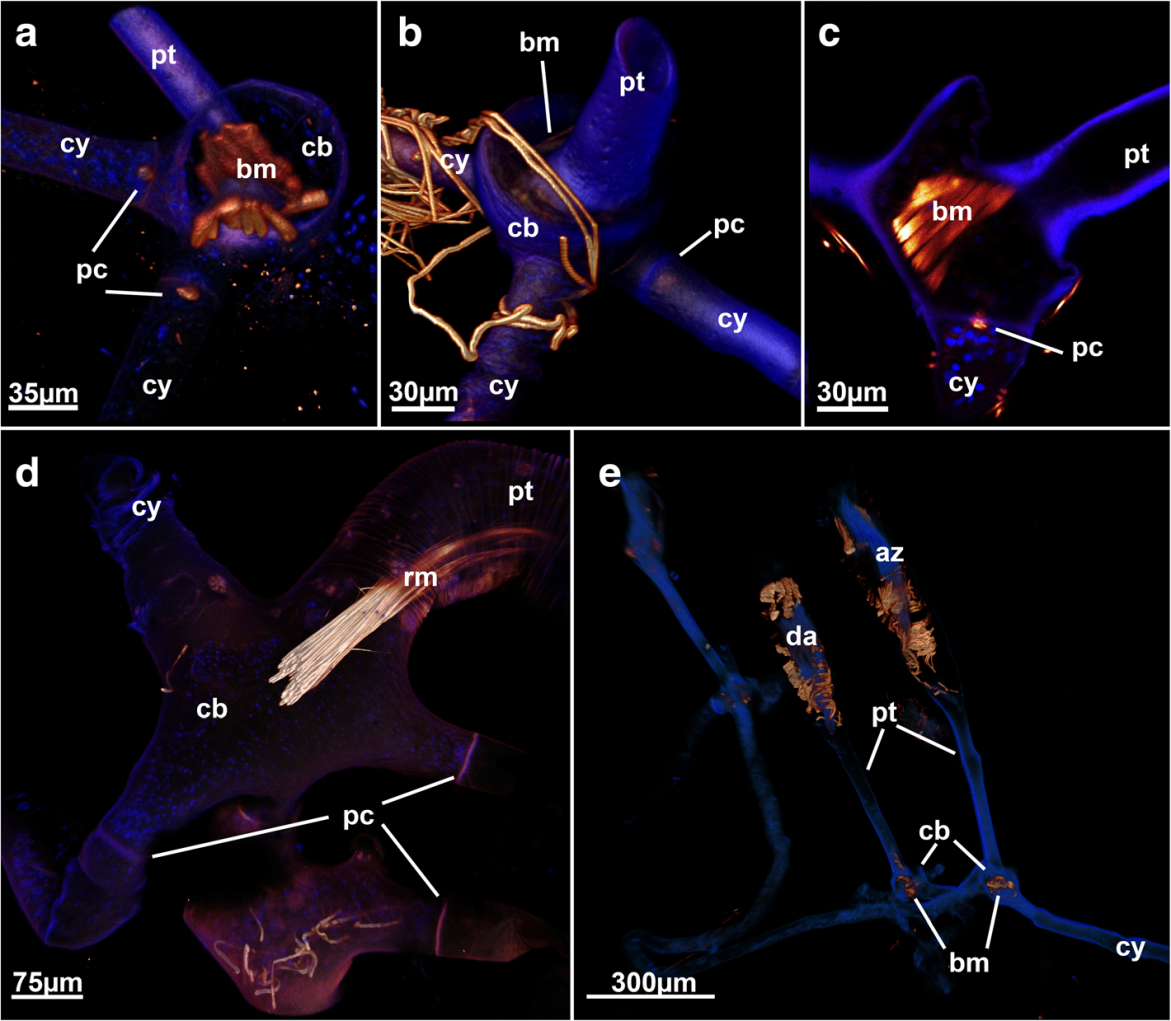
Proximal cystid muscles in the arachnidoidean *Nolella*(?) sp. **a** View of the proximal attachment side of cystids showing an enlarged base with distinct basal muscles. Volume rendering. **b** Similar such as found in (A) but different angle with cystid wall (blue) displayed with less transparency. Note the distinct swelling of the cystid base. Volume rendering. **c** Optical section through the enlarged cystid base showing the traverse of the basal muscle. **d** View of a cystid base with a thicker and more prominent peristomial tube. Note the lack of muscles in the wide cystid base. Volume rendering. **e** Lateral view of several zooids with thin peristomial tubes extending from the cystid base. Note that the left zooid is a developing bud. Abbreviations: az - autozooid, bm – basal muscle, cb – cystid base, cy – cystid appendage, da – developing autozooid, pc – pore complex, pt – peristomial tube, rm – retractor muscle

**Fig. 10:**
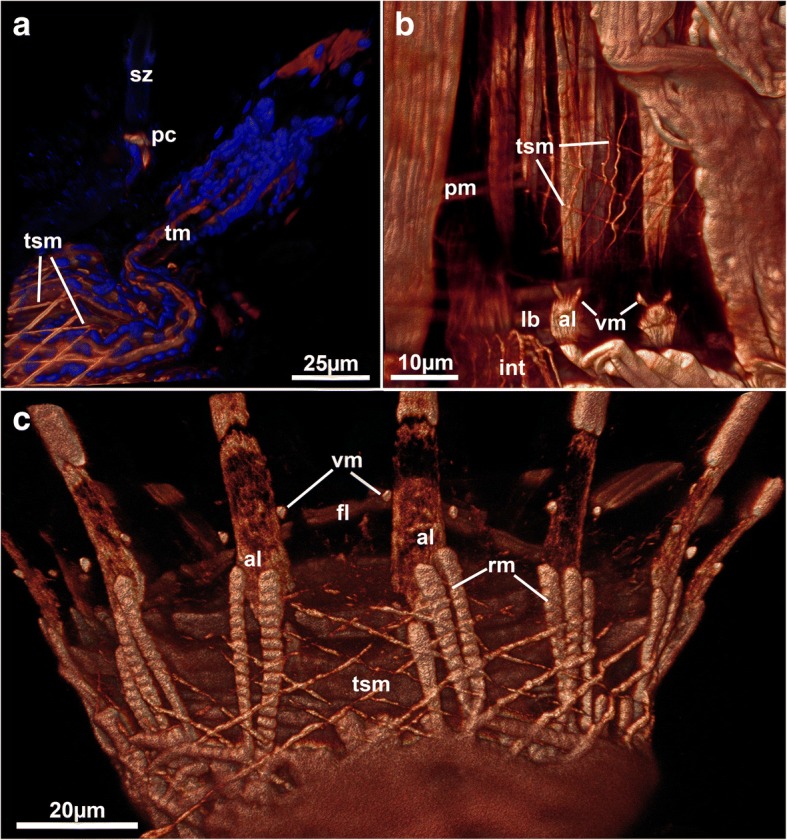
Tentacle sheath musculature in walkerioidean and victorelloidean ctenostomes showing a diagonal arrangement of muscle fibres. **a** Distal tip of the tentacle sheath of *Aeverillia setigera*. Note the pore complex separating the spinozooid. Volume rendering. **b** Lophophoral base of *Victorella pavida*. Maximum intensity projection. **c** Protruded lophophoral base of *Triticella flava*. Volume rendering. Abbreviations: al – abfrontal lophophoral base muscle, fl – frontal lophophoral base muscle, lb – lophophoral base, pc – pore complex, pm – parietal muscles, rm – retractor muscle, sz – spinzooid, tm – tentacle muscle, tsm – tentacle sheath muscles, vm – ‘v’shaped lophophoral base muscles

The pore complexes between adjacent zooids show distinct f-actin staining (Figs.3a, d, 5a, c, 7d, 8a, b, 9a-c and 10a). The f-actin concentration is asymmetrically distributed on both sides of the pore complexes. Medially, the signal passes through the thin pore bordered by the cystid cuticle where sometimes a distinct strong signal is found (Fig. 6a). Similar radial rosette-like muscles have been found in the pore complexes of *Hislopia malayensis* [28]. Ultrastructural investigations characterized rosette pore plates into three different categories: limiting cells, special cells and cincture cells [40–42]. Cincture cells are found adjacent to the cuticular pore, whereas special cells are dumbbell shaped with distinct larger portions on both sides of the pore [3, 41]. The encountered distribution of the f-actin signal in the analyzed ctenostomes corresponds to the dumbbell shaped morphology of the special cells. However, strong f-actin signal in the pore-bordering cincture cells, as is found in at least one a cheilostome bryozoan [31] is not entirely distinguishable.

In the cheilostome Gymnolaemata and a few ctenostomes, the pore complex is associated with funicular strands that connect the polypide with the communication pores [3, 42, 43]. Distinct connecting cords were rarely encountered in this study. Some cord-like structures were encountered in the f-actin stainings of *Victorella pavida* and *Nolella dilatata* (two genera with reported funicular interconnectivity, [11, 44]; Figs. 3d, 8b). In *Triticella flava*, which has thin proximal stalks, a thin tissue cord is present in some specimens. However, since no nuclei were encountered adjacent to the cystid cuticle (Fig. 8c), it appears that the encountered tissue cord is composed of the epidermis and possibly an underlying peritoneum. Consequently, previous reports of funicular cords in ctenostomes could in fact be parts of the body wall rather than distinct funicular strands.

Distinct funicular strands are present in some species in the form of peritoneal cords supplied with longitudinal musculature (Fig. 3). In *Paludicella articulata*, two funicular cords are present. Both extend from the caecum towards the body wall, one on the proximal side close to the tip of the caecum and a second more distally at the border of the pylorus (Fig. 3a). Most other encountered muscular funiculi were mostly singular and also extended from the caecum to the body wall (Fig. 3). This corresponds to the situation previously found in the ctenostome *Triticella minini* [35] and is also the general condition of Phylactolaemata and Stenolaemata [3]. Many cheilostomes possess a muscular caecal ligament in a similar area [45].

The Walkerioidea and Vesicularioidea produce polymorphic heterozooids in form of stolonal kenozooids [37]. Stolons consist of interconnecting cystids that possess the above mentioned pore complexes and linings of the cuticle. In contrast to the Vesicularioidea, stolons in walkerioideans possess distinct muscle bundles in their distal end close to the pore complexes (Fig. 5a and c). These bundles traverse the stolon from the basal to the frontal side and were sometimes referred to as ‘dorso-ventral muscles’ [37], but here will be referred to as stolonal transverse muscle. Walkerioideans possess large and elongated main stolons and shorter side stolons where the feeding autozooids are situated [37]. The stolonal transverse muscle is always present in the side stolon and fills almost the entire volume of the stolon (Fig. 5a). In the main stolon, it seems that the presence of this muscle depends on the colony form. In the creeping species *Mimosella verticillata*, the muscle is present in the main stolon (Fig. 5a) whereas in an erect species of the same genus, it is absent (Fig. 5c). Interestingly, the primary form of vesicularioidean ctenostomes is erect whereas walkerioideans are primarly creeping forms [37]. Vesicularioidean ctenostomes never show any stolonal muscles ([37, 42], this study), which, along with the lack of this muscle in the main stolon of the erect *Mimosella,* indicates that such a muscle is only functional and present in creeping forms. In the genus *Triticella* that only possess extremely small heterozooids as attachment sites only show very small bundles in these polymorphs (Fig. 7b and d).

**Fig. 11:**
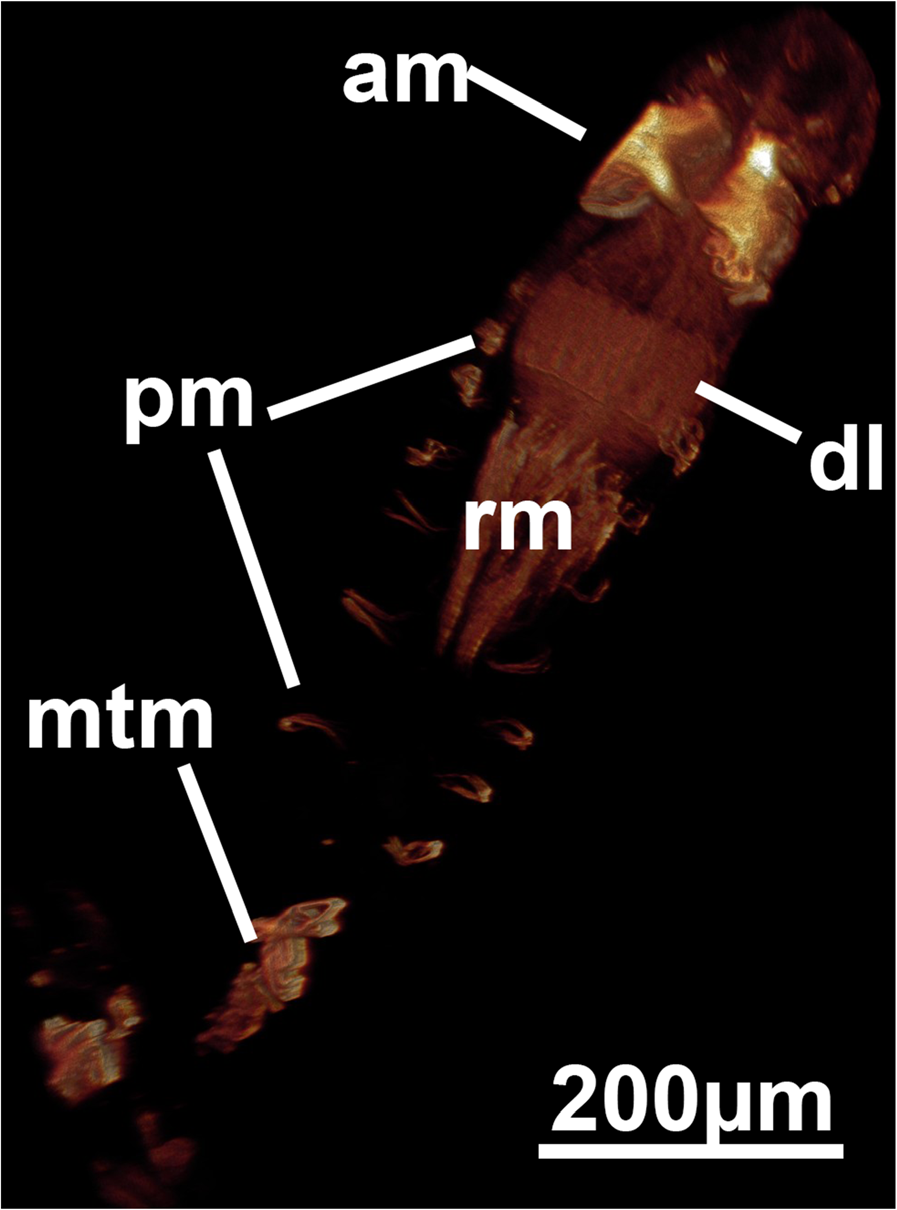
Developing bud of *Paludicella articulata* showing the transitory median transversal muscle that forms on the proximal side and later disappears during ontogeny. Abbreviations: am – apertural muscles, dl – developing lophophore, mtm – median transversal muscle, pm – parietal muscle, rm – retractor muscle

The arachnidioidean genus *Nolella* lacks any stolonal polymorphs and is characterized by very short cystid bases and thin appendages attached to the substrate. A very elongated peristomial tube extends from these basal appendages and contains the polypide [11, 39]. In one of the studied species distinct musculature similar to the stolonal transverse muscles of the walkerioideans was found in several zooids (Fig. 9a, c and e). The presence of this muscle appears to be correlated to younger zooids as it was more frequently encountered in smaller zooids with thin peristomial tubes and a broad cystid base (Fig. 9a, c and e), whereas larger zooids did not show any of these basal muscles (Fig. 9d). The presence and function of this muscle remains enigmatic. Since no other previously analyzed species of *Nolella* has any of these muscles (e.g [11, 46, 47]), the allocation of this species remains dubious. Accordingly, we designated this species as ‘*Nolella*(?)’ because a taxonomic discussion and revision of an already very difficult genus would be beyond of the scope of this study. Transitory basal muscles, present in this species are also found in budding stages of *Paludicella articulata* [48] (Fig. 11) in which young buds possess a transitory median transverse muscle close to the pore complex (Fig. 11) that is reduced during zooidal ontogeny [48]. This transitory muscle was considered to be homologous to the transverse stolonal muscle of walkerioideans. The temporary (?) muscle found in the *Nolella*(?) species could also represent a homologous structure (see also below).

#### Apertural musculature

Recently, ctenostome apertural muscles were reviewed in detail [28]. The present study confirms three principal sets of muscles: distal muscular fibers that are present as parieto-vestibular and as parieto-diaphragmatic muscles, second, peritoneal (parietovaginal) bands supplied with muscles, and the muscles associated with the vestibular wall (Fig. 2a).

**Fig. 12:**
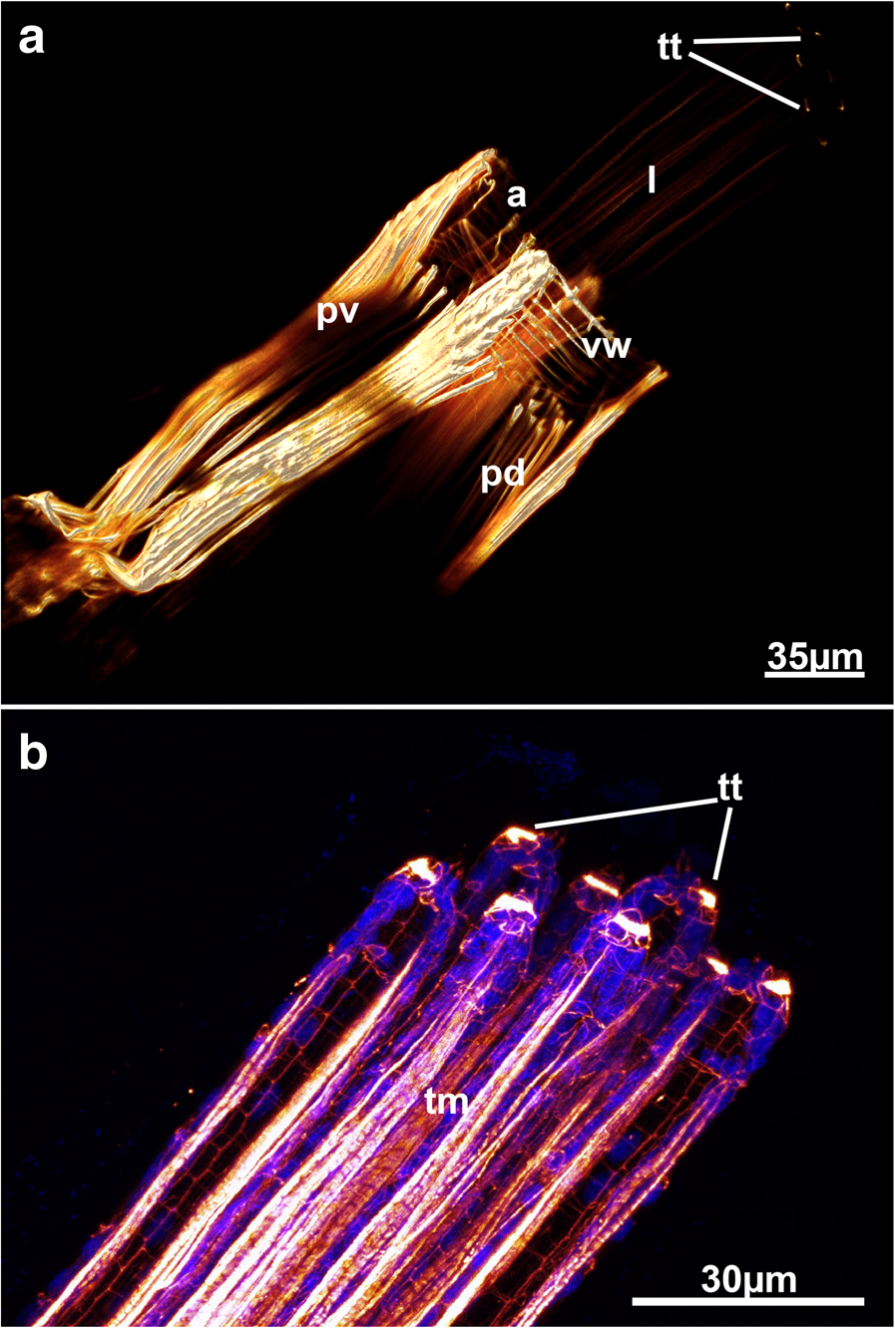
Myoanatomy of the apertural area with partially protruding lophophore of *Victorella pavida*. **a** Overview of the distal zooidal area with the tentacles protruding out of the aperture. **b** Detail of the tentacles showing the tentacle muscle bands and distal small sphincters at the tentacle tips. Abbreviations: a – aperture, l – lophophore, pd – parieto-diaphragmatic muscle, pv – parieto-vestibular muscle, tm – tentacle muscle, tt – tentacle tip, vw – vestibular wall

The distal parieto-diaphragmatic muscles are similar among most analyzed species (Fig. 6a-e and 12a) and run from the lateral body wall towards the diaphragm, the junction of the tentacle sheath and the vestibular wall. The diaphragm is supplied with a diaphragmatic sphincter, which closes the connection from the interior atrium to the vestibule (Figs. 4b, 6a-c and e-g). This sphincter is also present in the Phylactolaemata and Stenolaemata [28]. Distal to the diaphragm lies the vestibulum, which in most genera (*Nolella*, *Victorella*, *Paludicella*, *Mimosella*, *Amathia*, *Cryptopolyzoon*) has circular muscles that line the wall. Longitudinal muscle fibers are very rare in the vestibular wall. The condition in *Hislopia malayensis* is slightly different where a regular grid of circular and longitudinal muscles is present, but also diagonal muscles on its basal wall are found [28]. The alcyonidioid genera *Alcyonidium* and *Ascorhiza* lack any muscle over the entire range of the vestibular wall. Instead, a distally located prominent orifical sphincter is located that seals the vestibulum (Fig. 6e-g). The parieto-vestibular muscles are located more distally than the parieto-diaphragmatic muscles and connect the lateral body wall to the distal vestibular wall.

The parieto-vaginal bands are homologous to the duplicature bands of the Phylactolaemata [28, 30]. As concluded from all the data available, parieto-vaginal bands are present in the ctenostome clades Alcyonidioidea, Arachnidioidea, and Paludicelloidea (cf. [28], this study), whereas all Hislopioidea and most Walkerioidea, Vesicularioidea and Victorelloidea have reduced these muscular bands (see [28] for a recent comparison). With the exception of the Hislopioidea, representatives of the other major clades commonly form colonies with very elongated peristomes or even heteromorphic colonies with interconnecting stolons between feeding autozooids [37, 48]. Consequently, it was suggested that the loss of parieto-vaginal bands might be correlated with distinct stolonization of colonies or highly enlarged peristomes (cf. [28]). However, the lack of parieto-vaginal bands in hislopioideans and also the discovery of the parieto-vaginal bands in the arachnidioidean genus *Nolella* (Fig. 6a), the vesicularioidean genus *Cryptopolyzoon* (Fig. 4b) and the walkerioidean *Triticella minini* [35] shows that there is no distinct correlation. Noteworthy, parieto-vaginal bands were not encountered in *T. flava* in contrast to *T. minini*.

As indicated above, some variation exists in the general arrangement of these muscle groups. Most species have an arrangement of four parieto-diaphragmatic muscles, parieto-vestibular muscles and parieto-vaginal bands. This original arrangement is modified in some encrusting sheet-like runners such as *Hislopia malayensis* [28] or *Flustrellidra* [49], which show a bilateral arrangement of the apertural muscles with two large lateral portions. The alcyonidioid genera *Alcyonidium* and *Ascorhiza* do not have a distinct arrangement of clearly defined bundles such as other ctenostomes but possess a series of diffuse apertural muscle that extend from the body wall towards the vestibular wall (Fig. 6e, f). In addition, the stolon-bearing *Triticella flava* also has a bilateral arrangement of its apertural muscles. This species only has a single pair of parieto-vestibular muscles.

Some other genera also show slight modifications from the four parieto-diaphragmatic and vestibular muscles: one species of *Mimosella* has only two parieto-diaphragmatic muscles (but four parieto-vaginal ones, Fig. 6d). In *Paludicella articulata*, an additional bundle of a few muscle fibers is present that originates from the disto-lateral body wall and inserts proximally into the tentacle sheath (Fig. 6b and c). These muscle are arranged in parallel to the parieto-vaginal bands. These bundles appear to be unique for this species.

#### Tentacle sheath muscles

The ctenostome grade tentacle sheath generally shows longitudinal smooth muscle bands that are equally distributed over the entire range of the tentacle sheath, as found in the ctenostome *Hislopia malayensis* [28] and other gymnolaemates (Fig. 4b; [3]). In the analyzed walkerioidean and victorelloidean species investigated so far, the tentacle sheath muscles are comprised of diagonally orientated muscles instead of longitudinal fibers (Fig. 10). Given the wide distribution of only longitudinal muscles in other groups such as cheilostomes (e.g. [3]), cyclostomes [36] and some phylactolaemates [6, 30], it is clear that the diagonal basket of these two ctenostome families is derived. Depending on the phylogenetic interpretation and interrelations of the major ctenostome clades, this character might be an apomorphy for each of these clades or a synapomorphy (see also below).

#### Digestive tract musculature

**Fig. 13:**
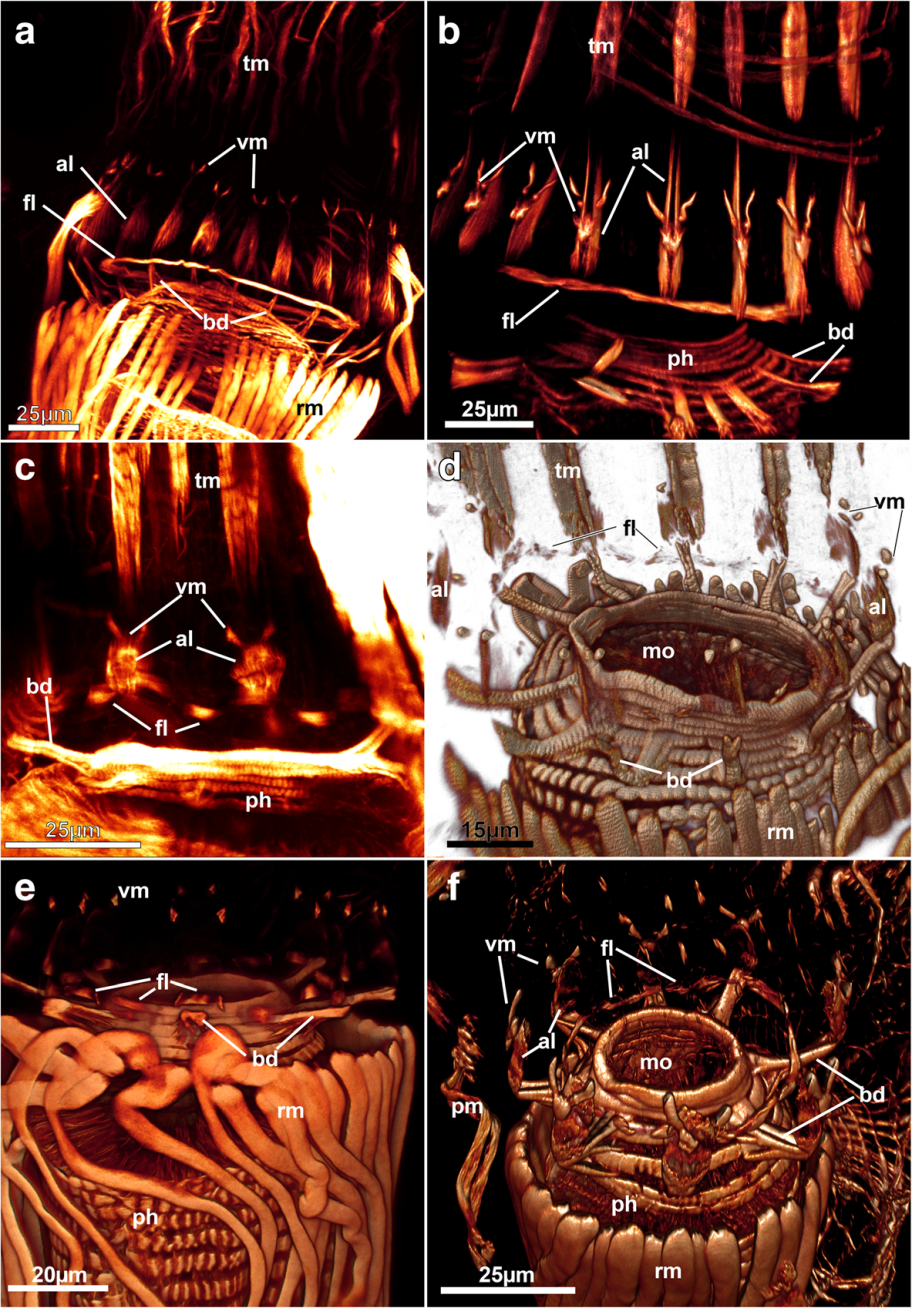
Myoanatomy of the lophophoral base of selected ctenostomes showing the four different sets of muscle in this region. **a** Arachnidioidea, *Nolella dilatata*. Lateral view, maximum intensity projection. **b** Paludicelloidea, *Paludicella articulata*. Lateral view, volume rendering. **c** Victorelloidea, *Victorella pavida*. Lateral view, maximum intensity projection. **d** Alcyonidioidea, *Alcyonidium gelatinosum*. Slight oblique view, volume rendering. **e** Vesicularioidea, *Amathia verticillata*. Lateral view, volume rendering. **f** Walkerioidea, *Mimosella verticillata*. Slight oblique view, volume rendering. Abbreviations: al – abfrontal lophophoral base muscle, bd – buccal dilatator, fl – frontal lophophoral base muscle, mo – mouth opening, ph – pharynx, rm – retractor muscle, tm – tentacle muscles, vm – ‘v-shaped’ lophophoral base muscle

The digestive tract among Bryozoa is divided into three distinct parts: foregut, stomach area and hindgut. The foregut consists of pharynx and esophagus. In all analyzed species these parts show a dense regular grid of cross-striated circular musculature (Figs. 2a; 3b, d; 13). The same condition is found among all other bryozoans [28, 30]. A few longitudinal muscle fibers were found in the foregut of a ctenostome ([28], Fig. 4a), a phylactolaemate [3] and a cyclostome [36]. The pharyngeal epithelium is present as a myoepithelium, i.e. the lateral cell borders are supplied with cross-striated actin filaments along with the triluminal pharyngeal space enabling this structure to function as suction pump [50]. This appears common among all non-phylactolaemates [36]. The other two areas of the digestive tract, the stomach area and the hindgut, mainly possess a more loose muscular basket as previously reported [28, 35]. The cardia has longitudinal as well as circular musculature (Fig. 4a). Several ctenostomes possess a prominent proventriculus or gizzard [51] used for mastication of food particles. Interestingly, concentrated ring muscles forming a cardiac sphincter have been observed in several species (Fig. 4b-e). Cardiac sphincters have been an essential character for species and genus discrimination among victorelloid ctenostomes [44]. The patchy distribution of a complex gizzard armed with distinct plates or teeth indicate that the gizzard evolved numerous times independently among ctenostomes [51]. Alternatively, it was suggested that a specialized sphincter at the cardia (in its most complex form a gizzard or muscular proventriculus) evolved once and was subsequently reduced in several ctenostomes lineages [37]. Our results support this notion by demonstrating the presence of a cardiac sphincter for the first time in an arachnidiodean species, the genus *Nolella* (Fig. 4c-e). A gizzard was suggested to be present in previous studies, but was never proven in the Arachnidioidea, which led to the assumption that it is absent ([52], see [37] for more detail). In summary, cardiac sphincters/proventriculus or a gizzard are found in Hislopioidea, some species of the Arachnidioidea, most Walkerioidea, Victorelloidea and all Vesicularioidea. This represents five of the seven main major clades of ctenostome bryozoans [8].

The caecum is supplied with a sparse net of mostly circular smooth muscles (Figs. 3b, d, 4a and e). A few longitudinal fibers are mainly found in the proximal end of the caecum. Some species show a more regular grid of circular and longitudinal muscles, but this seems to be the exception. As previously noted [28], this condition is strikingly different from the anatomy of Phylactolaemata where a dense cross-striated muscle basket is present [6, 30]. This is involved in peristaltic movements of food contents being pumped forwards and down within the caecum [5]. These movements are absent among Gymnolaemata and food particles are generally rotated in the gut lumen via ciliary action of the pylorus [53].

In non-phylactolaemates the remaining hindgut is separated by a pyloric region towards the intestine or rectum. This region is densely ciliated causing the active movement within the stomach [53]. As previously described for *H. malayensis*, the intestine terminating via the anus is supplied only with longitudinal smooth musculature in all species (Figs. 3d, 4a and 8b). This is again different from phylactolaemate species where only circular musculature is present (see [28]).

#### Lophophoral musculature

The lophophore is circular in all species, as is the case in all other non-phylactolaemates [3]. Several sets of muscles can be distinguished:

##### Lophophoral base muscles

Lophophoral base musculature varies in its appearance, but the basic setup of 3–4 sets of muscles as previously described for *Hislopia malayensis* is present in all species [28]. Proximally, several muscle bundles, the buccal dilatators, originate from the pharynx and traverse like spokes towards the lophophoral base (Fig. 2a; 13). These dilatators run at the base of each tentacle and thus correspond in number to that of the tentacles. In several instances, distinct striation is apparent in these fibers (Fig. 13d). Buccal dilatators have been found in all other studied gymnolaemate species (e.g. [3, 17]) and Cyclostomata [12, 36]. Approximately at the site where the dilatators insert; longitudinal muscle fibers extend on the lateral distal lophophoral side. These appear mostly paired and vary in the length from long (*Paludicella*, *Nolella*, ~ 25 μm, Fig. 13a and b) to rather short (e.g. *Victorella*, *Mimosella*, ~ 10-15 μm, Fig. 13c and f), which correlates with the general size of the autozooids. Sometimes these muscles appear only faintly in CLSM preparations. The muscles have so far only been described for the ctenostome *Hislopia malayensis* [28] and *Triticella minini* [35]. On its distal side or in some species even slightly above it, a set of previously described ‘v-shaped’ muscles (cf. [28]) represents the third set of lophophoral base muscles. In most species these do not appear as continuous ‘v’ such as found in *H. malayensis* [28], but are formed by three separate actin-rich elements: a median proximal element and two laterally and distally located ‘horns’ (Fig. 13b, d and f). In some species only the lateral parts are distinguishable with the median one being absent (Fig. 13a, c and e). The last and fourth muscle at the lophophoral base is located internally on the frontal side, approximately at the level where the buccal dilatators insert on their outer rim. This muscle constitutes a continuous ring of smooth muscles as previously reported for the hislopioidean *H. malayensis*, in arachnidioideans (*Nolella*, Fig. 13a) and paludicelloideans (*Paludicella*, Fig. 13b) [28]. Other major clades do not show a continuous ring musculature but similarly oriented, separate bundles of muscles (Fig. 13c-f). These are located between the tentacles and were first described in the cheilostome *Cryptosula pallasiana* as basal transversal muscle [17]. The distribution of these muscles in ctenostomes, however, is never as dense and thick such as found in the cheilostome *C. pallasiana* and often appears as an incomplete ring with bundles between the tentacles (Fig. 13c-f). This indicates that a continuous ring muscle is the possible ancestral state of this character. In the ancestral condition of cheilostomes and some ctenostomes it was subsequently reduced to several intertentacular muscle bundles. These intertentacular muscle bundles are much more pronounced in cheilostomes as basal transversal muscle.

##### Tentacle muscles

Tentacles in all species are always supplied with two longitudinal muscle bands – one on the frontal and another on the abfrontal side of each tentacle (Figs. 4b; 12b; 13b-d). This corresponds to the situation described for other bryozoans (Phylactolaemata: [3, 6, 20, 30, 36]; Cyclostomata: [36, 54]; Gymnolaemata: [16, 17, 28, 55]). Longitudinal tentacle muscles are commonly used for tentacle flicking [56], but also to contract the tentacles to make them more compact during the retraction process when contained within the tentacle sheath. Retracted tentacles are commonly contorted and their coelomic cavity often not directly visible due to the contracted state. The tentacle muscle bands are striated in all species. Only *Cryptopolyzoon wilsoni* has a different arrangement with striated abfrontal muscles and smooth frontal muscles. In *Triticella flava*, the frontal tentacle muscles start more distally than the abfrontal ones. However, samples of *Triticella flava* were preserved in a relaxed state with tentacles protruding from the orifice. Preservational differences may affect image interpretations when comparing well anesthetized samples to those where the lophophores are retracted.

In *Victorella pavida*, circular muscles were found on the distal tip of each tentacle (Fig. 12). Tentacles in bryozoans are generally the main organs for the dispersal of sperm [57, 58] and it appears likely that this species can actively open or at the very least close terminal tentacle pores for controlled release of sperm. A high-density of actin was also found in the tentacle tips of *H. malayensis* [28]. The actin concentrations in the tentacles of the latter, however, differ from those in *V. pavida* in not being sphincter-like muscles, but rather appear as pore-like structures on each tentacle. It is not clear why this muscle has not been found in any other species so far.

#### Retractor muscles

The retractor muscles are prominent muscle bands extending from the proximal or lateral body wall towards the lophophoral base and parts of the anterior digestive tract. Whether these muscles are smooth or striated has been discussed in previous works [3] whereas the current study supports both observations (Fig. 1; 3e; 4e; 7c; 9d; 13). Most species have smooth retractor muscle fibers similar to the situation of phylactolaemates [6, 30] and cyclostomes [36]. Previous data corroborate this finding [6, 28, 30, 35]. As mentioned, in some species retractor muscle fibers appear striated (Figs. 7c; 13d). The arrangement of the stripes appears too regular to reflect true arrangement of striated sarcomeres. In addition, the striation pattern is similar in retracted and protruded zooids. There appears to be a correlation between the number of retractor muscle fibres and these striation patterns: species with fewer and thicker fibres appear smooth whereas the striated appearance is common to species with fewer and thinner fibres. Other bryozoans have also been reported to contain striated compartments among retractor fibres (Phylactolaemata: *Fredericella sultana*, [30]; Ctenostomata: *Hislopia malayensis*, [28]). The significance and true nature of these striation patterns remains ambiguous and cannot be unambiguously answered by the methods employed to date. Nonetheless, it should inspire future work to analyze these patterns with transmission electron microscopy, particularly since it is now obvious that there exist differences among species.

### Myoanatomical characters in ctenostome phylogeny

Most previous phylogenetic analyses regarded flat uni- to multiserial encrusters (e.g. Hislopioidea, Alcyonidioidea) as early branching lineages mainly due to the similarity of fossil colony morphologies (see e.g. [8, 9]). The orifical area of these species is frontally located and in these forms is rather simple either being inconspicuously elevated from the remaining cystid wall or in form of an apertural/orificial papilla. These frontal elongations are generally called peristomes. Upright colonies such as those seen in the Paludicelloidea were considered to be a derived state given that the basic colony still retains an encrusting uni-serial colony type [37, 48]. Subsequent colony branches growing upright from the substratum to form erect, bushy colony types. The basic budding mode of one distal and two lateral buds is, however, retained in these colonies.

A second type of colony morphology is characterized by an elongation of the peristome that forms longs tubes. In most cases such as found in the Victorelloidea, the peristomes contain all the contents of the polypide and only the interconnecting proximal, cystidal parts of the zooids are on the basal side. In the Arachnidioidea, the various families and species show a high variation in peristomial length with e.g. some species of *Arachnidium* with short peristomes, or the family Nolellidae with extremely long peristomes (similar to victorelloideans) [37, 48].

A third group consisting of two ctenostome clades, the Vesicularioidea and Walkerioidea, have independtly formed kenzooids, polymorphs devoid of any polypides, that form interconnecting modules between feeding autozooids (‘trophons’ according to [37]). The mode of stolon formation, however, differs among these two major clades. The Walkerioidea form stolons by separating the peristomial tube from the proximal part whereas the peristome itself becomes the stolon in the Vesicularioidea. The latter invokes peristomial budding, i.e. buds being produced on the peristome area, a feature restricted to Victorelloidea and Vesicularioidea – hence their frequently assumed close relationship [37, 48].

We plotted our current results on proposed morphological phylogenies from Jebram [48] and Todd [8]. The latest tree of Waeschenbach et al. [59] was not considered, because of lack of representatives of any Victorelloidea, inclusions of derived forms of Walkeroidea (genus *Triticella*, see [37]), and the sole inclusion of a presumable arachnidioidean, *Anguinella palmata* (see also [37, 48]).

**Fig. 14:**
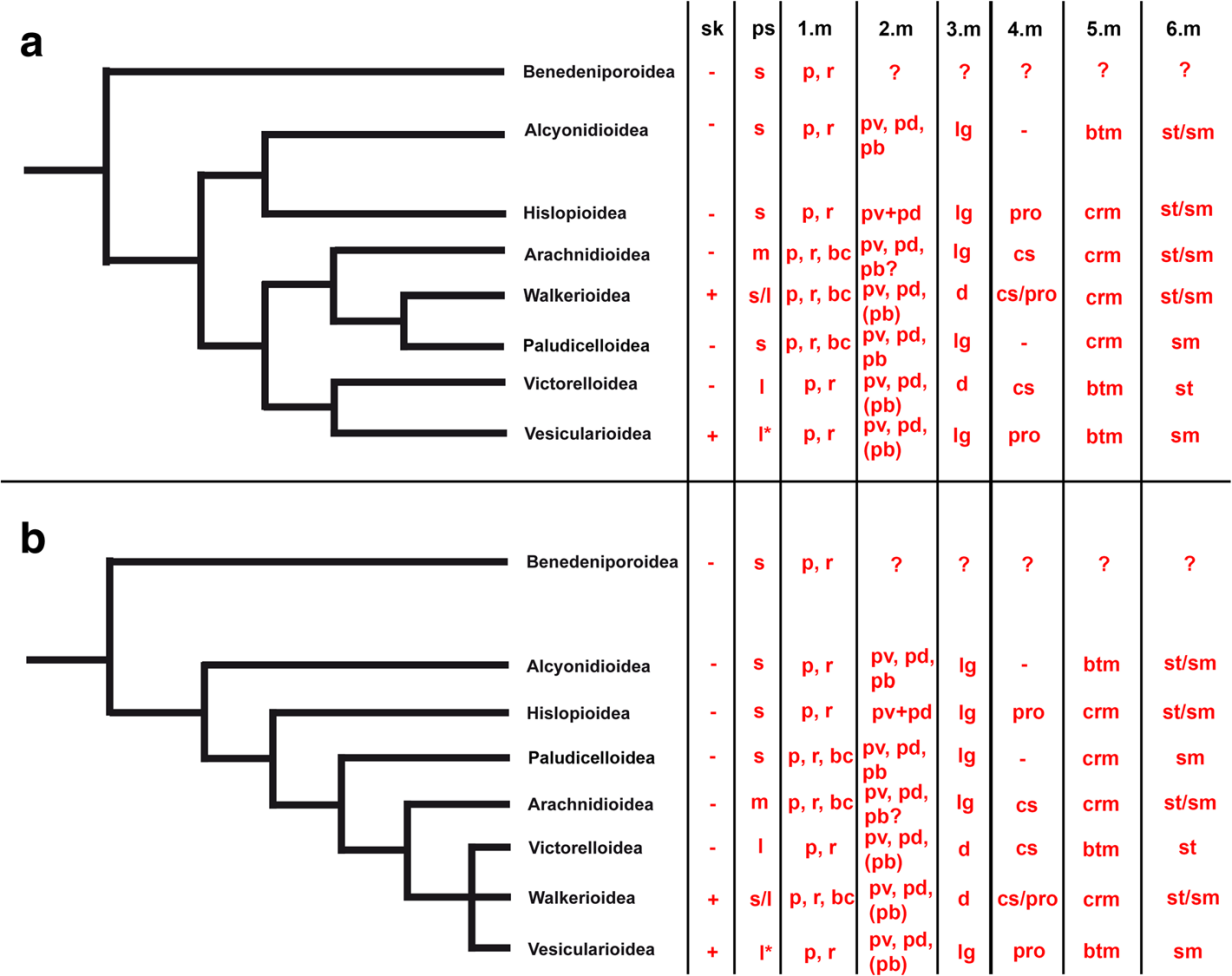
Phylogenetic reconstructions of Jebram 1986 [48] (**a**) and Todd 2000 [8] (**b**) including mapping of myoanatomical characters. First are listed the presence or absence of stolonal kenozooids (sk) and the size of the peristome (ps) followed by the six muscle sets described in this work. 1) body wall and derivates, 2) apertural muscles, 3) tentacle sheath muscles, 4) digestive tract muscles (here only cardia region mentioned, see text for details), 5) lophophoral base muscles, 6) retractor muscle. Abbreviations: bc – basal cystidal muscles (transitory? in Paludicelloidea and Arachnidioidea) or adult in stolons as stolonal transverse muscles (Walkerioidea), btm - basal transveral muscle of the lophophoral base, crm - continuous frontal ring muscle at the lophophoral base, cs – cardiac sphincter, d- diagonal tentacle sheath muscles, l – long persitome, l* - the long peristome forms the stolon in this group, lg – longitudinal tentacle sheath muscles, m – mixed (meaning both short and long peristomes are present), p – parietal muscles, pb – parietovaginal band, pd – parietodiaphragmatic muscle, ps – peristome, pro – proventriculus, pv – parietovestibular muscles, r - rosette-like pore complexes, s – short peristome, sk - stolonal kenozooids, sm – smooth retractor muscles, st – striated retractor muscles

According to the distribution of myoanatomical characters established by the current study, it remains difficult to determine which characters are plesiomorphic or apomorphic for each. Starting from the first muscle set, body wall and derivates, it is clear that parietal muscles are clearly plesiomorphic, but more interesting are the basal cystidial muscles restricted to the Walkerioidea, Arachnidioidea and Paludicelloidea (even though just transitory in the latter perhaps). According to Jebram’s interpretation (Fig. 14a), these three major clades have a monophyletic origin and are sister-group to a clade consisting of Victorelloidea and Vesicularioidea. According to the phylogenetic reconstruction of Todd (Fig. 14b), which also reconstructs a close-relationship of these five major clades, the basal cystidial muscles were most likely independently reduced in Victorelloidea and Vesicularioidea.

The second set, the apertural muscles, shows some variation among the different major clades, the most common being the reduction of the parieto-vaginal bands in some representatives of the major clades. As previously pointed out [28], stolonate forms appear to have reduced these bands. Comparison among all bryozoans show that these are present in the ground pattern and are plesiomorphic [28], hence calling for independent reduction of these structures in some ctenostomes.

The third set, tentacle sheath musculature is almost ubiquitously longitudinal with the exception of Victorelloidea and Walkerioidea. Depending on the phylogenetic reconstruction, these constitute independent, apomorphic characters according to Jebram’s tree (Fig. 14a) or possible synapomorphs according to Todd’s reconstruction (Fig. 14b).

Concerning the digestive tract musculature, only the cardiac sphincter/proventriculus shows a potential phylogenetically informative character (Table 2, Fig. 14). According to the two phylogenetic reconstructions, it appears likely that the cardiac sphincter was lost independently in both the Alcyonidioidea and Paludicelloidea. Alternatively, this feature may have evolved independently in a sister-group of the Alcyonidioidea comprising six major clades and was subsequently lost in the Paludicelloidea (Fig. 14). The apparent lack or very rare occurrence of such cardiac sphincters in cyclostome or cheilostome bryozoans [51] indicates that the group of ctenostomes closest to the cheilostome ancestor most likely lacked such a structure.

**Table 2:** List of specific muscular characters showing distinct variation among ctenostome bryozoans

Superfamily	Parieto-vaginal bands	Tentacle sheath	Cardia	Inner lophophoral base muscle	Retractor muscles
Alcyonidioidea	present	longitudinal musculature	not distinguished	separate, basal transverse muscle	smooth or striated
Arachnidioidea	present	longitudinal musculature	cardiac sphincter	continuous ring	smooth
Hislopioidea*	absent	longitudinal musculature	proventriculus	continuous ring	smooth (few striated parts recognized)
Paludicelloidea	present	longitudinal musculature	not distinguished	continuous ring	smooth
Victorelloidea	mostly absent	diagonal musculature	cardiac sphincter	separate, basal transverse muscle	smooth
Vesicularioidea	mostly absent	longitudinal musculature	proventriculus	separate, basal transverse muscle	smooth
Walkerioidea	mostly absent	diagonal musculature	cardiac sphincter or proventriculus	continuous ring	smooth or striated

*Data from current study or from Schwaha et al. 2011 [28]

The lophophoral muscles show high similarity among all ctenostomes. As indicated above, the inner lophophoral base muscles are arranged either as a single continuous ring or as distinct single basal transverse muscles. However, their distribution in the two phylogenetic trees does not seem to show any significant phylogenetic signal. Likewise, the distribution of the encountered retractor muscle fibre type (smooth vs. striated) does not seem to support certain nodes in the trees (Fig. 14).

## Conclusions

The present analysis demonstrates both the diversity and the similarities of the muscular systems of ctenostome bryozoans. It enlarges the dataset from two major clades to six. Differences in the main muscle systems are mainly present in the apertural muscle, arrangement of tentacle sheath muscles, presence of special cardiac musculature, the lophophoral base muscle or retractor muscles (Table 2). We have provided new numerous morphological data that will aid in unraveling character evolution of both ctenostome and gymnolaemate bryozoans. Our characters will be especially important for resolving character states among ctenostome bryozoans as this systematic classification represent a paraphyletic group [8]. Consequently, forthcoming analyses of additional morphological character evolution will not only yield insight in the ground pattern of ctenostomes, but also for gymnolaemates in general once a new robust phylogeny is available for ctenostomes.

## Additional file

Additional file 1: **Table S1.** List of muscular characters in the analysed ctenostome bryozoans. (DOCX 20 kb)

## References

[1] Bock P, Gordon DP (2013). Phylum Bryozoa Ehrenberg**,** 1831. Zootaxa.

[2] Hyman LH (1959). The invertebrates. Vol. V. Smaller coelomate groups.

[3] Mukai H, Terakado K, Reed CG, Harrison FW, Woollacott RM (1997). Bryozoa. Microscopic anatomy of invertebrates.

[4] Taylor PD, Larwood GP, Nielsen C (1981). Functional morphology and evolutionary significance of differing modes of tentacle eversion in marine bryozoans. Recent and fossil Bryozoa.

[5] Wood TS, Thorp JH, Rogers DC (2014). Phyla Ectoprocta and Entoprocta (bryozoans). Ecology and general biology, Vol I: thorp and Covich’s freshwater invertebrates, 4th edition.

[6] Gawin N, Wanninger A, Schwaha T (2017). Reconstructing the muscular ground pattern of phylactolaemate bryozoans: first data from gelatinous representatives. BMC Evol Biol.

[7] Nielsen C, Pedersen KJ (1979). Cystid structure and protrusion of the polypide in *Crisia* (Bryozoa, Cyclostomata). Acta Zool.

[8] Todd JA, Herrera Cubilla A, JBC J (2000). The central role of ctenostomes in bryozoan phylogeny. Proceedings of the 11th international Bryozoology association conference.

[9] Cheetham AH, Cook PL, Robinson RA (1983). General features of the class Gymnolaemata. Treatise on invertebrate paleontology part G: Bryozoa.

[10] Braem F (1890). Untersuchungen über die Bryozoen des süßen Wassers. Zoologica.

[11] Calvet L (1900). Contribution à l’histoire naturelle des Bryozaires Ectoproctes marins. Travaux de l’institut de zoologie de l’Université de Montpellier et de la station zoologique de Cette NS.

[12] Borg F (1926). Studies on recent cyclostomatous Bryozoa. Zool Bidr Uppsala.

[13] Gerwerzhagen A (1913). Untersuchungen an Bryozoen. Sitzungs Heidelb Akad Wiss Math Nat Kl Abt B.

[14] Graupner H (1930). Zur Kenntnis der feineren Anatomie der Bryozoen. Z Wiss Zool.

[15] Marcus E (1926). Beobachtungen und Versuche an lebenden Meeresbryozoen. Zool Jb Syst.

[16] Smith LW, Larwood GP (1973). Ultrastructure of the tentacles of Flustrellidra hispida (Fabricius). Living and fossil Bryozoa.

[17] Gordon DP (1974). Microarchitecture and function of the lophophore in the bryozoan Cryptosula pallasiana. Mar Biol.

[18] Gordon DP (1975). Ultrastructure and function of the gut of a marine bryozoan. Cah Biol Mar.

[19] Gruhl A, Bartolomaeus T (2008). Ganglion ultrastructure in phylactolaemate Bryozoa: evidence for a neuroepithelium. J Morphol.

[20] Gruhl A, Wegener I, Bartolomaeus T (2009). Ultrastructure of the body cavities in Phylactolaemata (Bryozoa). J Morphol.

[21] Shimizu K, Hunter E, Fusetani N (2000). Localisation of biogenic amines in larvae of Bugula neritina (Bryozoa: Cheilostomatida) and their effects on settlement. Mar Biol.

[22] Wanninger A, Koop D, Degnan BM (2005). Immunocytochemistry and metamorphic fate of the larval nervous system of Triphyllozoon mucronatum (Ectoprocta: Gymnolaemata: Cheilostomata). Zoomorphology.

[23] Santagata S (2008). The morphology and evolutionary significance of the ciliary fields and musculature among marine bryozoan larvae. J Morph.

[24] Santagata S (2008). Evolutionary and structural diversification of the larval nervous system among marine bryozoans. Biol Bull.

[25] Gruhl A (2008). Muscular systems in gymnolaemate bryozoan larvae (Bryozoa: Gymnolaemata). Zoomorphology.

[26] Gruhl A (2009). Serotonergic and FMRFamidergic nervous systems in gymnolaemate bryozoan larvae. Zoomorphology.

[27] Gruhl A (2010). Neuromuscular system of the larva of Fredericella sultana (Bryozoa: Phylactolaemata). Zool Anz.

[28] Schwaha T, Wood TS, Wanninger A (2011). Myoanatomy and serotonergic nervous system of the ctenostome Hislopia malayensis: evolutionary trends in bodyplan patterning of Ectoprocta. Front Zool.

[29] Schwaha T, Handschuh S, Redl E, Wanninger A (2015). Insights into the organization of plumatellid ‘larvae’ (Lophotrochozoa, Bryozoa) by means of 3D imaging and confocal microscopy. J Morphol.

[30] Schwaha T, Wanninger A (2012). Myoanatomy and serotonergic nervous system of plumatellid and fredericellid phylactolaemata (lophotrochozoa, ectoprocta). J Morphol.

[31] Schwaha TF, Wanninger A (2015). The serotonin-lir nervous system of the Bryozoa (Lophotrochozoa): a general pattern in the Gymnolaemata and implications for lophophore evolution of the phylum. BMC Evol Biol.

[32] Shunkina KV, Zaytseva OV, Starunov VV, Ostrovsky AN (2015). Comparative morphology of the nervous system in three phylactolaemate bryozoans. Front Zool.

[33] Temereva EN, Kosevich IA (2016). The nervous system of the lophophore in the ctenostome Amathia gracilis provides insight into the morphology of ancestral ectoprocts and the monophyly of the lophophorates. BMC Evol Biol.

[34] Ambros M, Wanninger A, Schwaha T (2018). Neuroanatomy of the plumatellid bryozoan Hyalinella punctata reveals a common pattern in a small group of freshwater bryozoans. J Morphol.

[35] Grischenko AV, Chernyshev AV (2015). Triticella minini - a new ctenostome bryozoan from the abyssal plain adjacent to the Kuril-Kamchatka trench. Deep Sea Res Part 2 Top Stud Oceanogr.

[36] Schwaha T, Handschuh S, Ostrovsky AN, Wanninger A. In Press. Morphology of the bryozoan *Cinctipora elegans* (Cyclostomata, Cinctiporidae) with first data on its sexual reproduction and the cyclostome neuro-muscular system. BMC Evolutionary Biology. 10.1186/s12862-018-1206-1 PMC6000935 29898669

[37] Jebram D (1973). Stolonen-Entwicklung und Systematik bei den Bryozoa Ctenostomata. Z zool Syst Evol.

[38] Schindelin J, Arganda-Carreras I, Frise E, Kaynig V, Longair M, Pietzsch T, Preibisch S, Rueden C, Saalfeld S, Schmid B (2012). Fiji: an open-source platform for biological-image analysis. Nat Methods.

[39] Hayward PJ (1985). Ctenostome bryozoans.

[40] Banta WC (1969). The body wall of cheilostome Bryozoa. II. Interzoidal communication organs. J Morphol.

[41] Gordon DP. Ultrastructure of communication pore areas in two bryozoans. In: Bryozoa, 1974. Pouyet S, vol. Doc. Lab. Geol. Fac. Sci. Lyon: Université Claude Bernard. H.S. 3; 1975. p. 187–192.

[42] Bobin G, Woollacott RM, Zimmer RL (1977). Interzooecial communications and the funicular system. Biology of bryozoans.

[43] Lutaud G. Autozooid morphogenesis in anascan cheilostomates. In: Treatise on invertebrates Palaeontology part G: Bryozoa (revised). Edited by Robinson RA, vol. Boulder: Geological Society of America. Soc Am. 1983;1:208–37.

[44] Braem F (1951). Über Victorella und einige ihrer nächsten Verwandten, sowie über die Bryozoenfauna des Ryck bei Greifswald. Zoologica.

[45] Lutaud G (1962). Sur la presence d'un muscle du caecum chez les bryozoaires chilostomes. Bull soc zool France.

[46] Rogick MD (1949). Studies on marine Bryozoa. IV. Nolella blakei n. sp. Biol Bull.

[47] Marcus E (1938). Bryozoarios marinhos brasileiros. II. Boletim da Faculdade de filosofia, ciências e letras, Universidade di Sao Paolo. Zoologia.

[48] Jebram D (1986). The ontogenetical and supposed phylogenetical fate of the parietal muscles in the Ctenostomata (Bryozoa). Z zool Syst Evol.

[49] Banta WC, Pouyet S (1975). Origin and early evolution of cheilostome Bryozoa. Bryozoa 1974.

[50] Nielsen C (2013). The triradiate sucking pharynx in animal phylogeny. Invertebr Biol.

[51] Markham JB, Ryland JS (1987). Function of the gizzard in Bryozoa. J Exp Mar Biol Ecol.

[52] Soule JD (1957). Two species of Bryozoa Ctenostomata from the Salton Sea. Bull South Calif Acad Sci.

[53] Silen L (1944). On the division and movements of the alimentary canal of the Bryozoa. Ark Zool.

[54] Nielsen C, Riisgard HU (1998). Tentacle structure and filter-feeding in Crisia eburnea and other cyclostomatous bryozoans, with a review of upstream-collecting mechanisms. Mar Ecol Prog Ser.

[55] Lutaud G (1973). L'innervation du lophophore chez le Bryozaire Chilostome Electra pilosa (L.). Z Zellforsch Mikroskop Anatom.

[56] Riisgard HU, Larsen PS (2010). Particle capture mechanisms in suspension-feeding invertebrates. Mar Ecol Prog Ser.

[57] Silen L (1966). On the fertilization problem in the Gymnolaematous Bryozoa. Ophelia.

[58] Ostrovsky AN (2013). Evolution of sexual reproduction in marine invertebrates: example of gymnolaemate bryozoans.

[59] Waeschenbach A, Taylor PD, Littlewood DTJ (2012). A molecular phylogeny of bryozoans. Mol Phylogenet Evol.

