# Supplementary material for: Unity in diversity: a survey of muscular systems of ctenostome Gymnolaemata (Lophotrochozoa, Bryozoa)
Source: Front Zool. 2018 Jun 7;15:24. doi: 10.1186/s12983-018-0269-6 (PMC5992719; doi:10.1186/s12983-018-0269-6)
Supplement: Supplementary file 1 — Table S1. List of muscular characters in the analysed ctenostome bryozoans. (DOCX 20 kb) [file 12983_2018_269_MOESM1_ESM.docx]

**Table S1: List of all studied ctenostome bryozoans and their muscle systems:**

**Abbreviations: 1) bc – basal cystid muscles, fm – flexing muscles, p – parietal muscles, r – rosette-like pore complex; 2) ds – diaphragmatic sphincter, os – orificial sphincter, pb – parietovaginal band, pd – parietodiaphragmatic muscle, pv – parietovestibular muscle, 3) d – diagonal muscles, lg – longitudinal muscles, 4) cs – cardiac sphincter, li – longitudinal muscles intestine, mp – muscular pharynx, pv – proventriculus, scm – smooth predominantly circular muscles, 5) al – abfrontal longitudinal muscles, bd – buccal dilatators, fl – frontal muscle as continuous ring muscle (crm) or separate basal transversal muscles (btm), vm – ‘v’-shaped muscles, 6) sm – smooth appearance, st – striated appearance.**

| **Superfamily** | **Family** | **Species** | **1. Body-wall & derivates** | **2. Apertural muscles** | **3. tentacle sheath muscles** | **4. digestive tract** | **5. lophophoral muscles** | **6. retractor muscles** |
| --- | --- | --- | --- | --- | --- | --- | --- | --- |
| Alcyonidioidea | Alcyonidiidae | *Alcyonidium gelatinosum* | p, r | *bilateral*, ds, os, pb, pd, pv | lg | mp, scm, li | al, bd, fl (btm), vm, | st |
| Alcyonidioidea | Alcyonidiidae | *Alcyonidium diaphanum* | p, r | *bilateral*, ds, os, pb, pd, pv, | lg | mp, scm, li | al, bd, fl (btm), vm, | st |
| Alcyonidioidea | Clavoporidae | *Ascorhiza* cf. *mawatarii* | ? | *bilateral*, ds, os, pb, pd, pv | lg | mp, scm, li | al, bd, fl (btm), vm, | sm |
| Arachnidioidea | Nolellidae | *Nolella dilatata* | p, r | ds,pb, pd, pv | lg | mp, scm, li  cs | al, bd, fl (crm), vm, | sm/st |
| Arachnidioidea | Nolellidae | *Nolella* sp. | p, r | ds, pb, pd, pv | lg | mp, scm, li  cs | al, bd, fl (crm), vm, | sm |
| Arachnidioidea | Nolellidae | *Nolella*(?) sp. | p, r ,bc | ds, pb, pd, pv | lg | mp, scm, li  cs | al, bd, fl (crm), vm, | sm |
| Paludicelloidea | Palludicellidae | *Paludicella articulata* | p, r ,bc? | *bilateral*, ds, pb, pd, pv | lg | mp, scm, li | al, bd, fl (crm), vm, | sm |
| Vesicularioidea | Vesiculariidae | *Amathia semiconvoluta* | p, r | ds, pd, pv | lg | mp, scm, li  pro | al, bd, fl (btm), vm, | sm |
| Vesicularioidea | Vesiculariidae | *Amathia (Zoobotryon) verticillata* | p, r | ds, pd, pv | lg | mp, scm, li  pro | al, bd, fl (btm), vm, | sm |
| Vesicularioidea | Buskiidae | *Cryptopolyzoon wilsoni* | p, r | ds, pb, pd, pv | lg | mp, scm, li  pro | al, bd, fl (btm), vm, | sm |
| Victorelloidea | Victorellidae | *Victorella pavida* | p, r | ds, pd, pv | d | mp, scm, li  cs | al, bd, fl (btm), vm, | st |
| Walkerioidea | Mimosellidae | *Mimosella gracilis* | p, r ,bc, fm | ds, pd, pv | d | mp, scm, li  pro | al, bd, fl (crm), vm, | sm |
| Walkerioidea | Mimosellidae | *Mimosella* sp. erect form | p, r ,bc, fm | ds, pd, pv | d | mp, scm, li  pro | al, bd, fl (crm), vm, | sm |
| Walkerioidea | Aeverilliidae | *Aeverillia setigera* | p, r ,bc | ds, pd, pv | d | mp, scm, li  pro | al, bd, fl (crm), vm, | st |
| Walkerioidea | Triticellidae | *Triticella flava* | p (single), r ,bc | *bilateral,* ds, pd, pv | d | mp, scm, li | al, bd, fl (crm), vm, | st |
